# Limb linkage rehabilitation training-related changes in cortical activation and effective connectivity after stroke: A functional near-infrared spectroscopy study

**DOI:** 10.1038/s41598-019-42674-0

**Published:** 2019-04-17

**Authors:** Congcong Huo, Gongcheng Xu, Zengyong Li, Zeping Lv, Qianying Liu, Wenhao Li, Hongzhuo Ma, Daifa Wang, Yubo Fan

**Affiliations:** 1 Beijing Key Laboratory of Rehabilitation Technical Aids for Old-Age Disability, National Research Center for Rehabilitation Technical Aids, Beijing, 100176 China; 2 Key Laboratory for Biomechanics and Mechanobiology of Ministry of Education, School of Biological Science and Medical Engineering, Beihang University, 100086 Beijing, China; 3 Rehabilitation Hospital, National Research Center for Rehabilitation Technical Aids, Beijing, 100176 China; 4 Key Laboratory of Rehabilitation Aids Technology and System of the Ministry of Civil Affairs, Beijing, 100176 China; 5 Beijing Advanced Innovation Center for Biomedical Engineering, Beihang University, Beijing, 100083 China

**Keywords:** Neuro-vascular interactions, Stroke, Stroke, Neuro-vascular interactions

## Abstract

Stroke remains the leading cause of long-term disability worldwide. Rehabilitation training is essential for motor function recovery following stroke. Specifically, limb linkage rehabilitation training can stimulate motor function in the upper and lower limbs simultaneously. This study aimed to investigate limb linkage rehabilitation task-related changes in cortical activation and effective connectivity (EC) within a functional brain network after stroke by using functional near-infrared spectroscopy (fNIRS) imaging. Thirteen stroke patients with either left hemiparesis (L-H group, n = 6) and or right hemiparesis (R-H group, n = 7) and 16 healthy individuals (control group) participated in this study. A multichannel fNIRS system was used to measure changes in cerebral oxygenated hemoglobin (delta HbO_2_) and deoxygenated hemoglobin (delta HHb) in the bilateral prefrontal cortices (PFCs), motor cortices (MCs), and occipital lobes (OLs) during (1) the resting state and (2) a motor rehabilitation task with upper and lower limb linkage (first 10 min [task_S1], last 10 min [task_S2]). The frequency-specific EC among the brain regions was calculated based on coupling functions and dynamic Bayesian inference in frequency intervals: high-frequency I (0.6–2 Hz) and II (0.145–0.6 Hz), low-frequency III (0.052–0.145 Hz), and very-low-frequency IV (0.021–0.052 Hz). The results showed that the stroke patients exhibited an asymmetric (greater activation in the contralesional versus ipsilesional motor region) cortical activation pattern versus healthy controls. Compared with the healthy controls, the stroke patients showed significantly lower EC (*p* < 0.025) in intervals I and II in the resting and task states. The EC from the MC and OL to the right PFC in interval IV was significantly higher in the R-H group than in the control group during the resting and task states (*p* < 0.025). Furthermore, the L-H group showed significantly higher EC from the MC and OL to the left PFC in intervals III and IV during the task states compared with the control group (*p* < 0.025). The significantly increased influence of the MC and OL on the contralesional PFC in low- and very-low-frequency bands suggested that plastic reorganization of cognitive resources severed to compensate for impairment in stroke patients during the motor rehabilitation task. This study can serve as a basis for understanding task-related reorganization of functional brain networks and developing novel assessment techniques for stroke rehabilitation.

## Introduction

Stroke is a prevalent neurological condition that remains the leading cause of long-term disability worldwide^1,2^. Approximately one-third of stroke patients experience a permanent motor deficit that impacts their daily living activities^3,4^. At present, a combination of task-specific training therapies remains the gold standard treatment for poststroke motor rehabilitation^3^. Recovery of function after stroke is a dynamic process involving neuronal reorganization^5^ that may occur in both the ipsilesional and contralesional hemispheres^6^. Therefore, an in-depth understanding of the mechanisms underlying rehabilitation task-related changes in the brain will guide advances in effective therapeutic interventions and prognostic indicators for stroke rehabilitation.

Limb linkage rehabilitation training can stimulate motor function in the upper and lower limbs simultaneously and allow coordinated movement of the upper and lower limbs. In respective training tasks, reaching movements of the upper and pedaling movement patterns of the lower limbs are performed. During movement of the upper and lower extremities using limb linkage rehabilitation training, patients can enhance muscle strength and coordination of their upper and lower limbs by functional exercise training. Evidence shows that motor rehabilitation can improve motor recovery in stroke patients, but brain function and reorganization patterns related to the motor rehabilitation tasks in upper and lower limb linkage have not been well understood. Measures of brain activity during a dynamic motor rehabilitation task may provide important information to elucidate the cortical mechanisms involved in motor rehabilitation. Conventional functional imaging tools, such as functional magnetic resonance imaging (fMRI) and positron emission tomography (PET), are unsuitable for such measurement because they are vulnerable to patient motion and are very confined at their measuring facilities. In recent years, interest in the use of functional near-infrared spectroscopy (fNIRS) to assess brain function and understand brain-behavior interactions has increased. fNIRS is a neuroimaging technique that provides noninvasive detection of relative changes in cerebral oxygenated hemoglobin (delta HbO_2_) and deoxygenated hemoglobin (delta HHb) at the cortical surface^7^. It is an optical imaging method based on hemodynamic responses as an indirect measure of neural activation^8^. This method has been widely used to monitor changes in cerebral hemodynamics during rehabilitation tasks in stroke patients^9–11^. Compared with fMRI and PET, fNIRS is relatively robust with regard to patient motion and is also a safe, portable and low-cost method for monitoring brain activity^12^. Accordingly, fNIRS is suitable for measuring brain activity and investigating brain reorganization patterns during motor rehabilitation tasks^13,14^.

Recent developments in neuroscience have emphasized that the brain is organized into a set of widely distributed networks that play a fundamental role in controlling behavior^15–17^. Focal injuries in the brain after stroke can have remote effects on the functional network architecture of cortical areas in both hemispheres^18–22^. Previous studies have demonstrated that the cortical reorganization underlying neurological deficits can be better assessed over entire networks by using a connectivity-based method^23,24^. This method can provide insights into the network reorganization of the brain and has important implications for behavior and recovery^25–27^. Functional interactions based on neuroimaging data can be described in two ways: (i) functional connectivity (FC) and (ii) effective connectivity (EC). FC is defined as the statistical dependencies among remote neurophysiological events^28^. It is based on correlations among measures of neuronal activity, but does not provide any direct insight into how correlations are mediated. Functional integration within a distributed network is usually better described in terms of EC, which refers explicitly to the influence of one neural system over another^28^. EC can provide crucial knowledge about the direction of information flow and facilitate interpretation regarding the causality of interactions among brain regions. Dynamic Bayesian inference (DBI) is a promising approach to exploit EC to determine the influence of one oscillator over another under a particular model of causal dynamics^29,30^. The information provided by EC allows us to investigate the specific role of a cortical region during a given task.

In this study, we aimed to investigate motor rehabilitation task with upper and lower limb linkage-related changes in EC patterns within a brain network in stroke patients. Previous studies have reported increased activation of the frontoparietal cortex^31^ and other nonmotor brain areas, such as the occipital cortex^32^, in association with motor tasks in stroke patients. In the present study, 24-channel fNIRS equipment was used to measure delta HbO_2_ and delta HHb in the prefrontal cortex (PFC), motor cortex (MC), and occipital lobe (OL). EC was calculated in these regions for each signal type (delta HbO_2_ and delta HHb) by using coupling functions and DBI based on a coupled-phase-oscillator model in stroke patients and healthy controls under different conditions. We hypothesized that EC patterns in stroke patients will change in association with performance of the motor rehabilitation task with upper and lower limb linkage. This study serves as a basis to understand mechanisms of neural rehabilitation and develop novel assessment techniques for stroke rehabilitation based on cortical fNIRS signals.

## Materials and Methods

### Participants

A total of 29 participants, including 13 stroke patients with either left hemiparesis (L-H group, n = 6) or right hemiparesis (R-H group, n = 7) and 16 age-matched healthy individuals with no history of psychiatric, orthopedic, or neurological diseases (control group) participated in this study. For stroke patients, the inclusion criteria were as follows: (1) 2 weeks from the onset of ischemic stroke; (2) unilateral supratentorial lesions; and (3) moderate to severe motor deficits in the hemiplegic side’s upper and lower extremities. The exclusion criteria were as follows: (1) any clinically significant or unstable medical disorder; and (2) any neuropsychiatric comorbidity other than stroke. The clinical characteristics of the stroke patients are shown in Table 1. The demographic data of the participants are shown in Table 2. Age, sex, and BMI were matched between the groups.

**Table 1:** Clinical details of stroke patients

Patients	Age	Sex	Handness	Time post stroke	Type of stroke	Hemiparesis side	Site of lesion	NIHSS	FMA-UE	FMA-LE	MMSE
Pt-1	62	M	R	17days	I	L	R interal carotid artery occlusion, R Frontal, temporal, parietal lobes	12	6	4	21
Pt-2	21	M	R	30days	I	L	R temporal lobe, parietal lobe, basal ganglia	3	12	20	29
Pt-3	54	M	R	1year	I	R	L thalamus, basal ganglia	4	25	20	30
Pt-4	52	M	R	180days	I	L	R frontal lobe, parietal lobe, temporal lobe	7	8	15	27
Pt-5	67	M	R	40days	I	R	L basal ganglia, lateral ventricle, occipital lobe	11	15	7	22
Pt-6	49	M	R	90days	I	L	L basal ganglia, parietal lobe	7	4	10	29
Pt-7	30	F	R	5year	I	R	L temporal lobe, parietal lobe	3	15	25	29
Pt-8	62	M	R	30days	I	L	R basal ganglia, thalamus	4	10	20	22
Pt-9	24	M	R	50days	I	R	L basal ganglia	10	8	10	16
Pt-10	69	F	R	60days	I	R	L basal ganglia, parietal lobe, thalamus Large atherosclerotic foci	11	6	5	19
Pt-11	61	M	R	15days	I	L	R basal ganglia artery stenosis, basal part of the pons, parietal lobe	5	5	8	28
Pt-12	46	M	R	90days	I	R	L basal ganglia, insula and lateral ventricle	6	16	9	29
Pt-13	57	M	R	180days	I	R	L frontal lobe, temporal lobe, parietal lobe, insula, basal ganglia, corona radiata infarction	14	5	8	25

Pt = patient; M = male; F = female; L = left; R = right; I = ischemic; NIHSS = NIH stroke scale; FMA-UE = Fugl-Meyer Assessment-upper extremities; FMA-LE = Fugl-Meyer Assessment-lower extremities; MMSE = Mini-mental State Examination.

**Table 2:** Participant characteristics (Mean ± SD presented).

	Controls (N = 16)	Stroke Patients		Between group *p* value		
		R-H (N = 7)	L-H (N = 6)	*p* ^*a*^	*P* ^*b*^	*P* ^*c*^
Age (years)	55.9 ± 8.1	49.6 ± 17.3	51.2 ± 15.8	0.270	0.431	/
Gender (M/F)	9/7	5/2	6/0	0.418	0.067	/
BMI	25.2 ± 3.5	25.4 ± 3	25.6 ± 3	0.886	0.793	/
Systolic blood pressure(mmHg)	130.6 ± 11.5	134.3 ± 22.4	136.3 ± 20.7	0.625	0.474	/
Diastolic blood pressure(mmHg)	76.3 ± 11.3	80 ± 11.3	78 ± 5.4	0.435	0.729	/
MMSE	26.7 ± 2.4	24.3 ± 5.5	26 ± 3.6	0.148	0.690	0.526
Time post stroke	/	376.7 ± 660.5	60.3 ± 64.7	/	/	0.270
NIHSS	/	8.4 ± 4.1	6.3 ± 3.2	/	/	0.334
FMA-UE	/	12.7 ± 6.8	7.5 ± 3.1	/	/	0.112
FMA-LE	/	12.6 ± 7.9	12.8 ± 6.6	/	/	0.950

*p*^*a*^ for the difference between control group and R-H group.

*p*^*b*^ for the difference between control group and L-H group.

*P*^*c*^ for the difference between R-H group and L-H group.

Experiments were conducted with the informed and written consent of each participant. The experimental procedure was approved by the Human Ethics Committee of National Research Center for Rehabilitation Technical Aids and was in accordance with the ethical standards specified by the Helsinki Declaration of 1975 (revised in 2008).

### Experimental procedures

**Figure 1:**
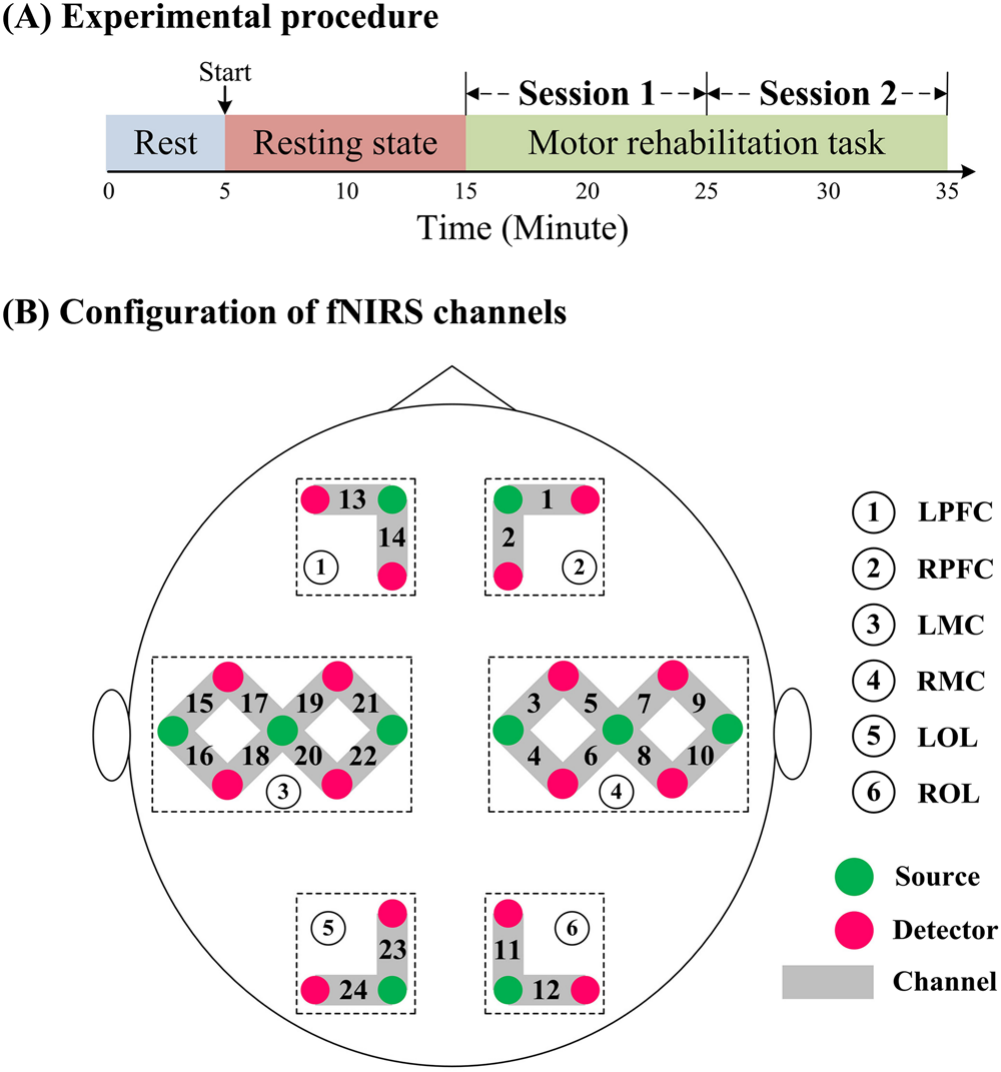
(**A**) Experimental procedure. There are three states in this study, which are the resting, task_S1 and task_S2 state, respectively. (**B**) The configuration of fNIRS channels. The pink dots represent light detectors and the green dots represent light sources. 10 sources and 16 detectors result in 24 channels, covering six brain regions: LPFC, RPFC, LMC, RMC, LOL and ROL.

Before the experiment began, the participants were allowed to rest for 5 min in a quiet environment to relax. After resting, they were instructed to maintain a comfortable sitting position, remain still, and relax with eyes closed for 10 min in a quiet, dimly lit room (resting state). The participants were reminded to stay awake and then perform the motor rehabilitation task. Before the task was performed, the limb linkage rehabilitation training device (Model: REX7000, SCIFIT, USA) was checked to ensure it was in normal working order. After checking to confirm the safe function of the seat, a therapist helped each participant to sit and then adjusted the armrest, pedal, and backrest according to the patient’s needs. Comfort of the participants during rehabilitation training was ensured. The paretic limbs of stroke patients were immobilized with bandages to ensure safety during motion. Uniform training intensity was applied to all rehabilitation training task samples. During the task, participants were asked to perform rehabilitation training of the upper and lower limbs linkage at a voluntary speed for 20 min. Motor rehabilitation was under active conditions. The training process was guided by a professional therapist. The first 10 min of the motor rehabilitation task was defined as Session 1 (task_S1) and the last 10 min as Session 2 (task_S2). The experimental procedure is shown in Fig. 1(A).

### fNIRS measurement

The fNIRS system (NirScan Danyang Huichuang Medical Equipment Co. Ltd., China) with wavelength of 740, 808, and 840 nm was used to detect the cerebral hemodynamic changes during the resting state and rehabilitation task state. Data was sampled with a frequency of 10 Hz. The distance between the detector and the source was 30 mm to ensure propagation to the gray matter beneath the optodes^33,34^. In this study, the fNIRS channels were defined as the midpoint of the corresponding light source-detector pairs. A total of 24 channels were built by 10 light sources and 16 detectors for fNIRS measurement. These channels were symmetrically distributed in the left and right hemispheres of the participants (12 channels on each side) and positioned based on the 10/10 international electrode placement system. The location of these channels covered the left and right PFC (LPFC/RPFC), left and right MC (LMC/RMC), and left and right OL (LOL/ROL), as shown in Fig. 1(B). The calibration function of the instrument and corresponding template were used to ascertain the channels to fill exactly in correspondence of the 10/10 electrode positions with the different head size of the participants. Each optode was attached to the skull surface by using a custom-made hard plastic cap and covered with a black cloth to prevent penetration of ambient light.

### Data preprocessing

**Figure 2:**
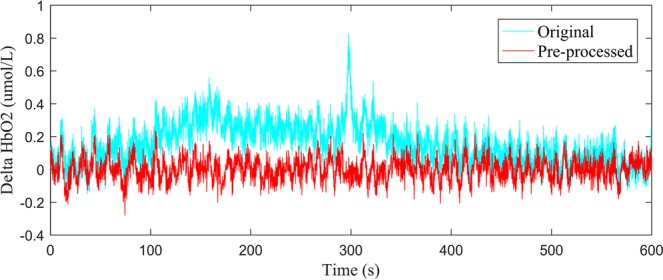
Delta HbO_2_ signals before and after pre-processing.

Fluctuations in concentrations of delta HbO_2_ and delta HHb were calculated from changes in detected light intensity according to the modified Beer-Lambert law, assuming constant scattering^35^. Data preprocessing was performed after delta HbO_2_ and delta HHb signals were obtained. We used the moving average method to eliminate the obvious abnormal points in the signal. The time window used for the moving average filter was 3 s. A processing method based on moving standard deviation and cubic spline interpolation was then applied to remove motion artifacts^36^. Artifacts were distinguished by identifying the sliding window standard deviation above a certain threshold and were removed by cubic spline interpolation. Finally, we used a six-order Butterworth bandpass filter of 0.021–2 Hz to obtain filtered signals with an improved signal-to-noise ratio. More details about the filter can be found in the Supplement 1 (Supplemental Methods and Materials section). The time series with respect to delta HbO_2_ before and after pre-processing are shown in Fig. 2.

### Wavelet transform

Continuous wavelet transform (WT), which can project signals from the time to the time-frequency domain, enable us to continuously derive the frequency content in time by adjusting the length of wavelet windows^37^. They are a scale-independent method in terms of frequency. The WT of a signal *g*(*u*) is then defined as

1 $$g(s,t)={\int }_{-\infty }^{\infty }{{\rm{\Psi }}}_{s,t}(u)\cdot g(u)du$$

For our analysis, we used the Morlet wavelet as the mother wavelet. Morlet mother wavelets belong to Nonorthogonal wavelet function and it is a complex plane wave multiplied by a Gaussian envelope function^38^. The rationale for using the Morlet wavelet is that its Gaussian envelope provides good localization of events in both time and frequency^39^. s is the scaling factor. To create various scales of the wavelet comparable to the original signal, the mother wavelet is stretched and compressed by s, which can be converted easily to the corresponding frequency *f*. Due to the complex nature of the Morlet wavelet, wavelet coefficients for each time *t*_*n*_ and frequency *f* obtained by WT are defined as:

2 $${g}_{k}(f,t)={a}_{k}(f,{t}_{n})+i{b}_{k}(f,{t}_{n})$$

From here the instantaneous phase can be determined as

3 $${{\rm{\Phi }}}_{k}(f,{t}_{n})=\arctan ({b}_{k}(f,{t}_{n})/{a}_{k}(f,{t}_{n}))$$

In this study, oscillators of delta HbO_2_ and delta HHb signals in four major frequency-specific intervals were distinguished by using WT as follows^37^: high-frequency bands (HF; I, 0.6–2 Hz; II, 0.145–0.6 Hz), low-frequency band (LF; III, 0.052–0.145 Hz), and very-low-frequency band (VLF; IV, 0.021–0.052 Hz).

### Effective connectivity analysis

The dynamical interactions between a pair of oscillators can be described by the form of the corresponding coupling function^40,41^. The form of the coupling function has recently attracted considerable attention as a means to describe the physical rule specifying how the functionality of the interactions occur, more than just a way of investigating correlations and statistical effects. A widely used approach for studying coupling functions between interacting oscillators is through their phase dynamics^42,43^. A coupled-phase-oscillator model between each channel pair was established according to the phase dynamic information obtained from the original signal by WT. DBI was applied to extract the optimal set of parameters describing the model^30^. This method is capable of isolating the specific influence of each oscillator on the others in the model^44,45^. Importantly in this context, the methods used for the reconstruction of coupling functions belong to the group of EC techniques.

Based on the phase dynamics of the oscillators derived from the chosen frequency intervals, we consider a model of two coupled phase oscillators^46^, described by the stochastic differential equation, and it is defined as^30^:

4 $${\dot{\varphi }}_{t}(t)={w}_{i}(t)+{q}_{i}({\varphi }_{i},{\varphi }_{j},t)+{\xi }_{i}(t)$$

with *i* ≠ *j* and *i*, *j* = {1, 2}. *w*_*i*_(*t*) is the parameter for the natural frequency, and the *ξ*_*i*_(*t*) is considered to be Gaussian white noise. The function *q*_*i*_ of the two oscillators’ phases *ϕ*_*i*_ and *ϕ*_*j*_ represents the coupling configuration. The deterministic periodic part of (4) can be Fourier-decomposed into a sum of base functions, the phase dynamics ${\dot{\varphi }}_{i}(t)$ can be rewritten as:

5 $${\dot{\varphi }}_{i}(t)=\sum _{K=-K}^{K}{c}_{k}^{(i)}{{\rm{\Phi }}}_{k}({\varphi }_{i},{\varphi }_{j},t)+{\xi }_{i}(t)$$

In this study, we used a second-order Fourier expansion (*K* = 2). The Fourier components Φ_*k*_ act as base functions for the DBI to evaluate the parameters ${c}_{k}^{(i)}$ for describing the couplings. The parameters *c*_*k*_ can be calculated recursively using the equations listed in the paper^47,48^. Once we have the inferred parameters *c*_*k*_, we can calculate the coupling quantities and characteristics. Coupling parameter ${\sigma }_{i,j}$ is defined as Euclidean norm of the inferred parameters from the phase dynamics:

6 $${\sigma }_{i,j}=\sqrt{\sum _{k=-K}^{K}{({c}_{k}^{(i:j)})}^{2}}$$

Coupling parameters can provide a quantitative measure of the information flow between the coupled systems including the coupling strength (CS) and coupling direction. The value of σ_*i,j*_ provides an overall estimate of the influence of oscillation *i* on oscillator *j*.

### Surrogate test

A surrogate test was applied to ascertain whether the detected coupling parameters are genuine or spurious. Amplitude-adjusted Fourier transform (AAFT) surrogate signals were generated by shuffling the phases of the original time series to create new time series with the same means, variances, autocorrelation functions as the original sequences, but without their phase relations^49^. This approach should have removed any dependence or causal relationship, if present in the original data, while preserving the basic statistical properties of the original data^50^. The coupling parameters evaluated for the surrogate signals should then reflect a “zero level” of the coupling relationship^41^. We averaged 100 coupling parameters calculated from surrogate signals, and considered that coupling parameters from the original recordings were statistically significant when they were 2 standard deviations above the mean surrogate coupling values. Details on the generation of surrogate signals can be found in Supplement 1 (Supplemental Methods and Materials section). In this study, the frequency-specific coupling parameters for each signal type (delta HbO_2_ and delta HHb) of the 24 channels were calculated based on the coupled-phase-oscillator model and DBI for each participant in different conditions. The significant channel-wise ECs were obtained for each channel pair to describe the directed interactions among the channels.

### Region-wise effective connectivity

In this study, we reported the results of motor rehabilitation task-related changes in EC patterns with respect to delta HbO_2_ and delta HHb signals to further investigate mechanisms of neural rehabilitation in stroke patients. To more clearly characterize EC patterns among the six brain regions, significant coupling parameters derived from all possible pairs of 24 channels were averaged as over ${{\rm{A}}}_{6}^{2}=30$ directed interregional connectivity types, thus corresponding to mutual interactions among the six regions: LPFC→RPFC, LPFC→LMC, LPFC→RMC, LPFC→LOL, LPFC→ROL, RPFC→LPFC, RPFC→LMC, RPFC→RMC, RPFC→LOL, RPFC→ROL, LMC→LPFC, LMC→RPFC, LMC→RMC, LMC→LOL, LMC→ROL, RMC→LPFC, RMC→RPFC, RMC→LMC, RMC→LOL, RMC→ROL, LOL→LPFC, LOL→RPFC, LOL→LMC, LOL→RMC, LOL→ROL, ROL→LPFC, ROL→RPFC, ROL→LMC, ROL→RMC, ROL→LOL (region 1→region 2, region 1 was the source of directed influence and region 2 was the target of the directed influence).

### Statistical analysis

The characteristic values of age, BMI, blood pressure, MMSE, NIHSS, FMA-UE, FMA-LE and gender were expressed as the mean (SD) or percentages. The Kolmogorov-Smirnov and Levene tests were applied to test the variance normality and homogeneity of the data at group level. Significant differences of the characteristic values were determined using *t*-test for means and SDs, and chi-square test for percentages. A two-way repeated-measures (ANOVA) was carried out for each signal type to test the significance of the effect of interest (main effect of group and condition and interaction of group × condition), where the group (R-H, L-H, and control groups) is a between-subject factor and the condition (resting state, task_S1, and task_S2) is a repeated-measures factor. The Bonferroni *t*-test was used for pair-wise comparisons. Three inter-condition comparisons (task_S1 vs. resting state; task_S2 vs. resting state; task_S2 vs. task_S1) were designed. Thus, the *α* value was adjusted as 0.0167 (0.05/3). One-way ANOVA was applied to identify statistically significant differences for in the coupling parameters between the patients and controls. Two inter-group comparisons (R-H group vs. control group; L-H group vs. control group) were designed. Thus, the *α* value was set as 0.025 (0.05/2).

## Results

### Cortical activation patterns during the rehabilitation task

**Figure 3:**
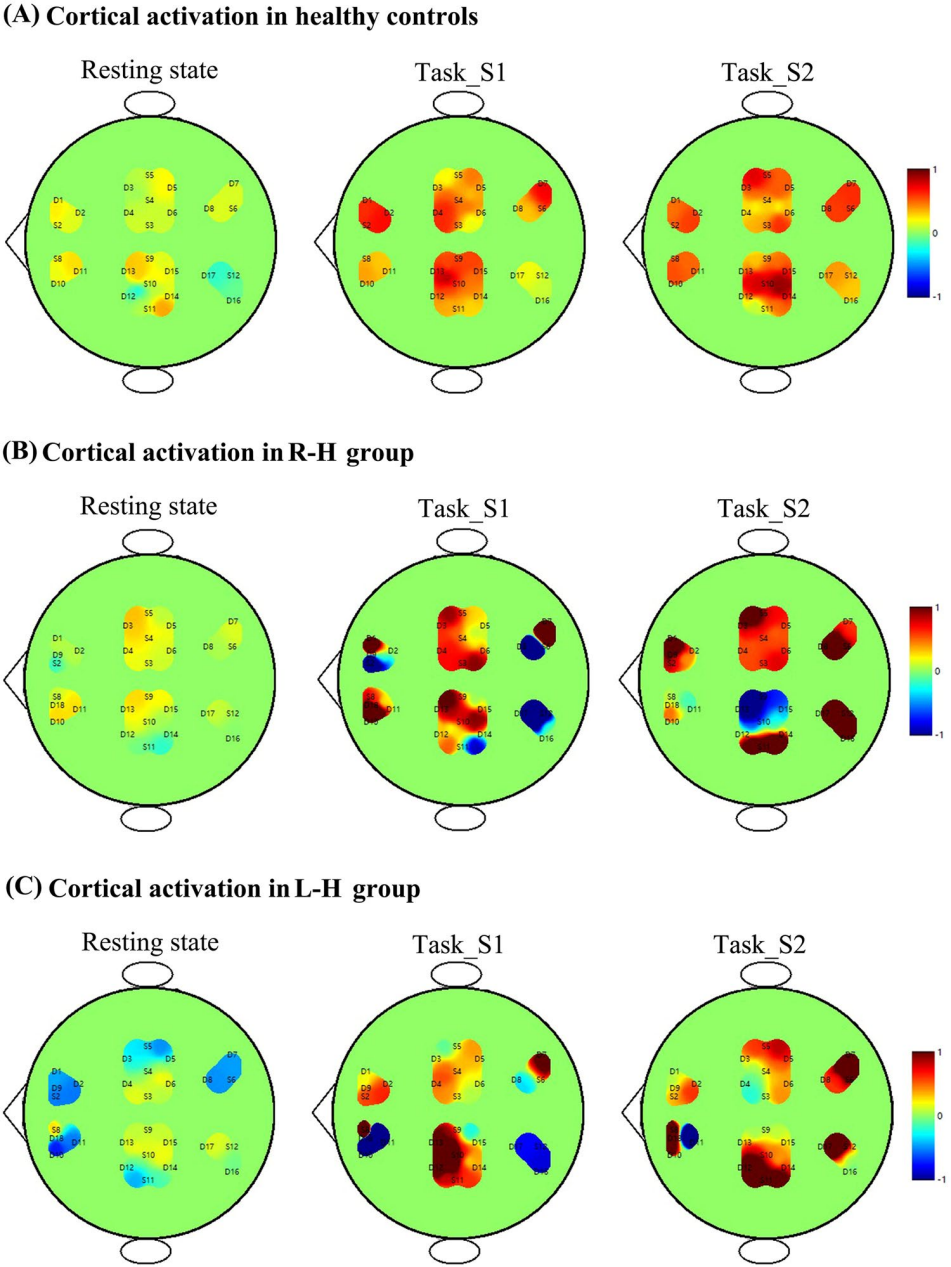
Cortical activation patterns with respect to delta HbO_2_ during different conditions in participants (**A**) healthy control; (**B**) patient with right-hemiparesis; and (**C**) patient with left-hemiparesis.

Figure 3 shows cortical activation patterns with respect to delta HbO_2_ in healthy controls and stroke patients during resting state, task_S1, and task_S2. The letters indicate the positions of the light source and detector, and a pair of adjacent light sources and detectors forms a channel. In a given state, the average value of the delta HbO_2_ concentration change (over 10 min) at the channel midpoint represents its change in the channel region, from which an image is generated by interpolating the inverse distance. The color bar number range on the right specifies the color depth. In healthy controls, an increase in delta HbO_2_ and a decrease in delta HHb were observed in bilateral motor areas during task states compared with the resting state. However, the increase in delta HbO2 (and decrease in delta HHb) in stroke patients was greater in the contralesional than that in the ipsilesional motor region during the rehabilitation task (Fig. 3 and Fig. S3). These results indicated that the stroke patients exhibited an asymmetric (greater activation in the contralesional versus ipsilesional motor region) cortical activation pattern compared with healthy controls.

### Effective connectivity

Repeated-measures ANOVA on the frequency-specific coupling parameters for delta HbO_2_ signals with the between-subject factor “group” and the within-subject factor “conditions” revealed that the CS values in interval I showed a significant main effect of group (*p* < 0.05) in the following connectivity: RPFC→LPFC, LPFC→LMC, LMC→LPFC, RMC→LPFC, LPFC→LOL, LOL→LPFC, RPFC→LMC, RMC→RPFC and RMC→LMC, and a significant main effect of condition in the LMC→RMC connectivity (*p* = 0.034). No significant interaction was found between the two factors. The CS in interval II showed a significant main effect of group in the LPFC→LOL connectivity (*p* = 0.0004). No significant effect of condition and interaction between the two factors was found in interval II. The CS values in interval III showed a significant main effect of group (*p* < 0.05) in the following connectivity: RMC→LPFC, LPFC→LOL, ROL→LPFC, RPFC→LMC, RPFC→LOL, LMC→RMC, RMC→LMC, LMC→LOL, LOL→LMC, ROL→LMC, RMC→LOL and ROL→LOL. No significant main effect of condition was found in this interval. There was significant group × condition interaction (*p* < 0.05) in interval III in the following connectivity: LMC→LPFC, RMC→LPFC and ROL→LPFC. The CS values in interval IV showed a significant main effect of group (*p* < 0.05) in the following connectivity: LPFC→RPFC, RPFC→LPFC, LMC→LPFC, RMC→LPFC, LOL→LPFC, ROL→LPFC, RPFC→LMC, LMC→RPFC, RMC→RPFC, LOL→RPFC, ROL→RPFC, LOL→LMC and LOL→RMC. No significant main effect of condition and group × condition interaction was found in this interval.

### Changes in EC from resting state to task state

**Figure 4:**
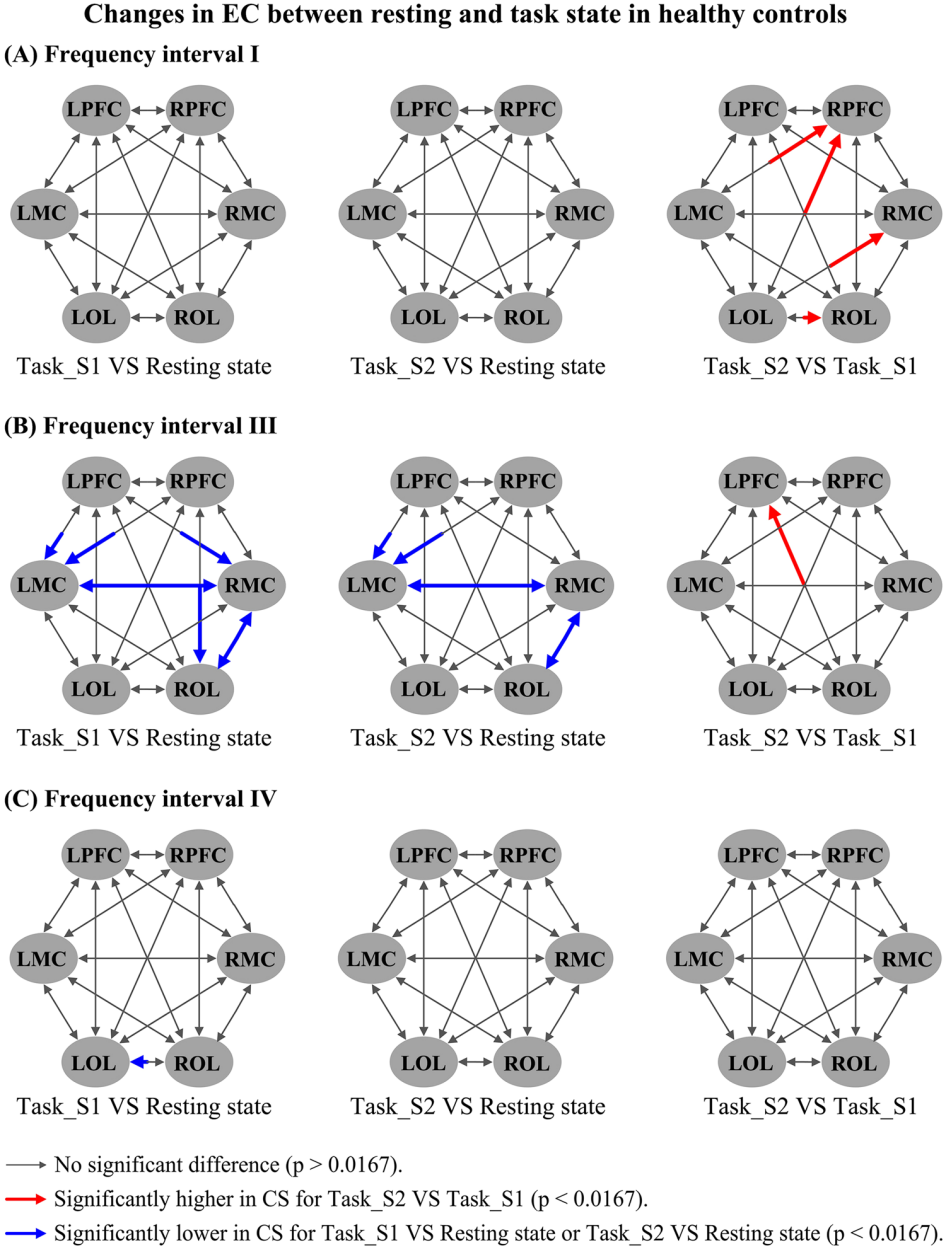
Changes in frequency-specific EC for delta HbO_2_ signals in the healthy controls between different states: (**A**) Comparison in frequency interval I; (**B**) Comparison in frequency interval III; (**C**) Comparison in frequency interval IV; Three comparisons are made between conditions: task_S1 VS resting state; task_S2 VS resting state; task_S2 VS task_S1; Comparison results are coded in arrow color and size, as illustrated by a legend.

**Figure 5:**
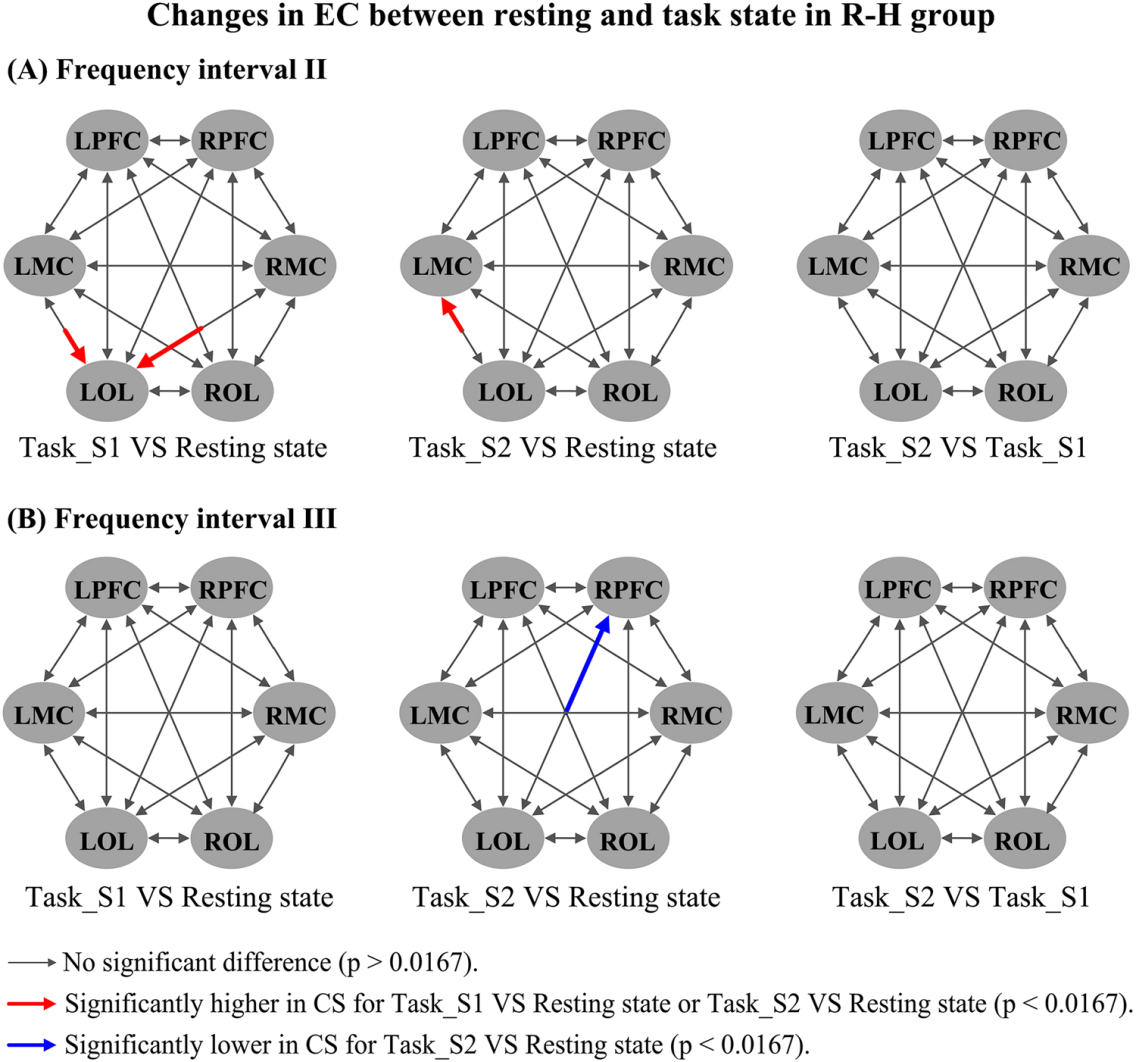
Changes in frequency-specific EC for delta HbO_2_ signals in the R-H group between different states: (**A**) Comparison in frequency interval II; (**B**) Comparison in frequency interval III; Three comparisons are made between conditions: task_S1 VS resting state; task_S2 VS resting state; task_S2 VS task_S1; Comparison results are coded in arrow color and size, as illustrated by a legend.

**Figure 6:**
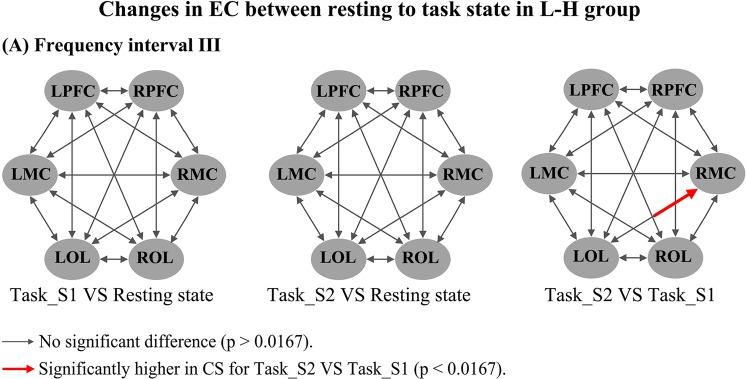
Changes in frequency-specific EC for delta HbO_2_ signals in the L-H group between different states: (**A**) Comparison in frequency interval III; Three comparisons are made between conditions: task_S1 VS resting state; task_S2 VS resting state; task_S2 VS task_S1; Comparison results are coded in arrow color and size, as illustrated by a legend.

Figure 4 shows the changes in frequency-specific EC with respect to delta HbO_2_ signals from the resting to task states in the healthy controls. The CS valves in interval III were significantly decreased in task_S1 compared with the resting state in the following connectivity: RMC→LMC (*p* < 0.002), LPFC→LMC (*p* = 0.002), LPFC→RMC (*p* = 0.01), RPFC→LMC (*p* = 0.002), LMC→RMC (*p* < 0.0001), ROL→RMC (*p* = 0.006), RMC→ROL (*p* = 0.004), and RPFC→ROL (*p* = 0.003). The CS valves in interval III were significantly decreased in task_S2 compared with the resting state in the following connectivity: RMC→LMC (*p* = 0.006), LPFC→LMC (*p* = 0.013), RPFC→LMC (*p* = 0.003), LMC→RMC (*p* = 0.001), ROL→RMC (*p* = 0.001), and RMC→ROL (*p* = 0.001). The CS valve in interval IV was significantly decreased in task_S1 compared with the resting state in the connectivity ROL→LOL (*p* = 0.014). Compared with that in task_S1, the CS valves in task_S2 were significantly increased in LOL→RPFC (*p* = 0.001), LOL→RMC (*p* = 0.005), LOL→ROL (*p* = 0.01), and LMC→RPFC (*p* = 0.009) in interval I and in ROL→LPFC (*p* = 0.012) in interval III. CS valves for delta HHb signals (Fig. S4) were significantly decreased in task states in intervals III and IV when compared with resting state. Moreover, the CS valves in intervals I and II in the task states were significantly higher than that those in the resting state (see Supplementary results for details). Figure 5 shows the changes in frequency-specific EC with respect to delta HbO_2_ signals from the resting to task state in the R-H group. Compared with the resting state, the CS valves in interval II were significantly increased in LMC→LOL (*p* = 0.016) and RMC→LOL (*p* = 0.005) in task_S1 and in LOL→LMC (*p* = 0.0015) in task_S2. The CS from LOL to RPFC (*p* = 0.014) in interval III was significantly decreased in task_S2 compared with the resting state. In the result of delta HHb signals (Fig. S5), compared with the resting state, the CS in interval III was significantly decreased from RMC to LPFC in task_S2 state. Significantly increased CS values were found in intervals I and II in task states compared with those in the resting state. Figure 6 shows the changes in frequency-specific EC with respect to delta HbO_2_ signals from the resting to task state in the L-H group. Compared with the task_S1, the CS in task_S2 was significantly increased in LOL→RMC (*p* = 0.001) in interval III. No significant difference in CS was found in L-H group between conditions in intervals I, II and IV. According to the result for delta HHb signals (Fig. S6), compared with the resting state, the CS in interval II was significantly increased in LOL→RMC in task_S2. In this interval, the CS was significantly lower in ROL→RMC in task_S2 compared with that in task_S1.

### Comparison of EC between healthy controls and stroke patients under different conditions

**Figure 7:**
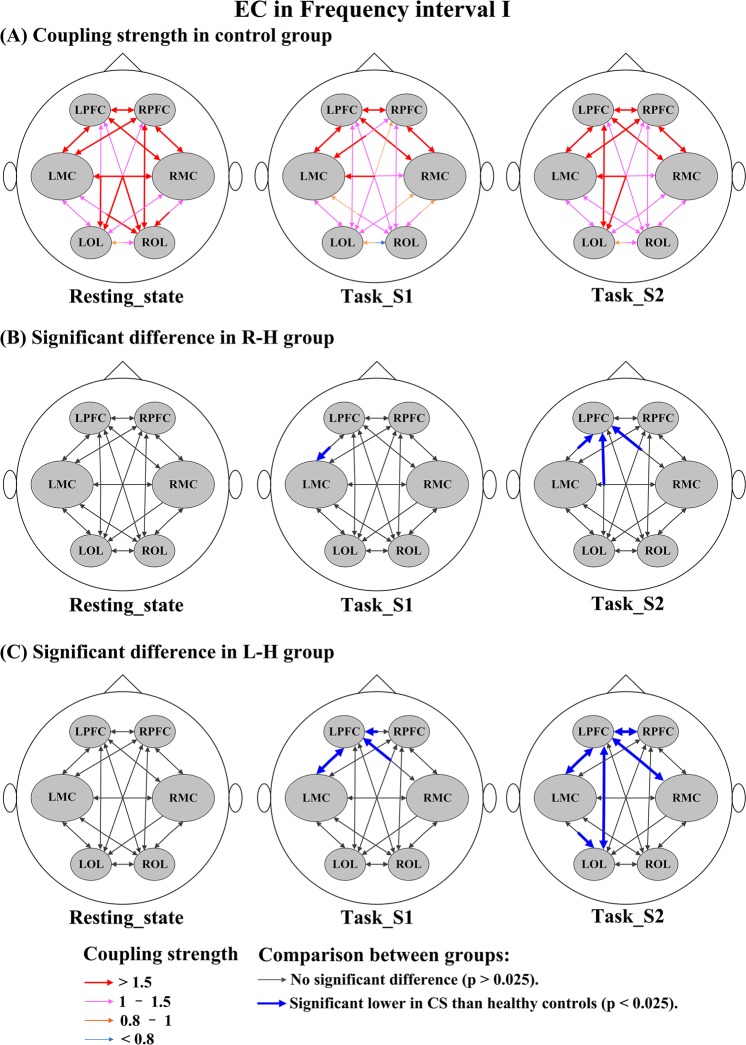
Changes in EC among brain regions for delta HbO_2_ signals in interval I under different conditions: (**A**) CS in control group under different conditions; Coupling parameters indicate the connection strength and direction, which are coded in the size and color of the arrows. (**B**) Significant difference in R-H group compared with healthy controls; (**C**) Significant difference in L-H group compared with healthy controls.

**Figure 8:**
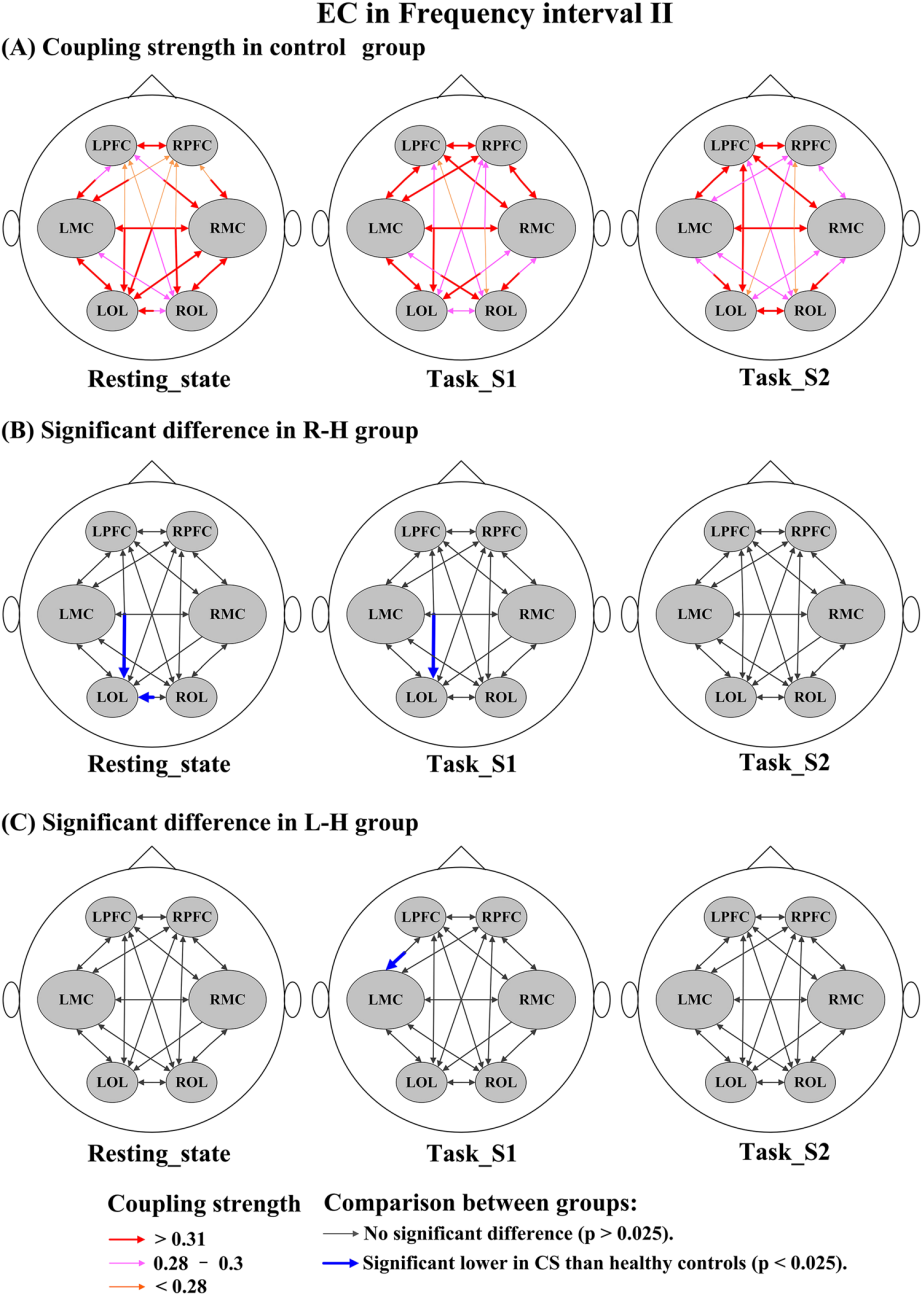
Changes in EC among brain regions for delta HbO_2_ signals in interval II under different conditions: (**A**) CS in control group under different conditions; Coupling parameters indicate the connection strength and direction, which are coded in the size and color of the arrows. (**B**) Significant difference in R-H group compared with healthy controls; (**C**) Significant difference in L-H group compared with healthy controls.

**Figure 9:**
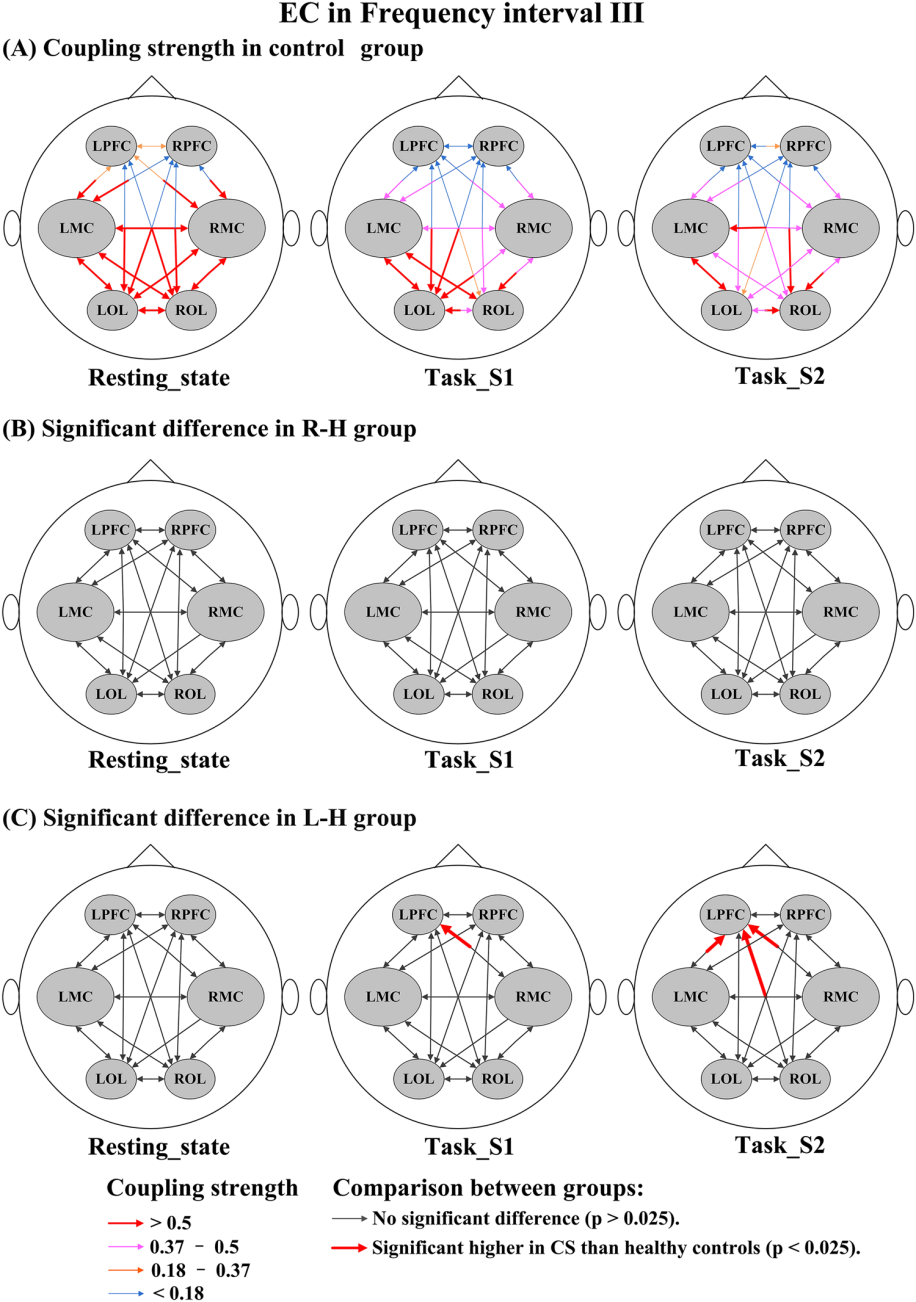
Changes in EC among brain regions for delta HbO_2_ signals in interval III under different conditions: (**A**) CS in control group under different conditions; Coupling parameters indicate the connection strength and direction, which are coded in the size and color of the arrows. (**B**) Significant difference in R-H group compared with healthy controls; (**C**) Significant difference in L-H group compared with healthy controls.

**Figure 10:**
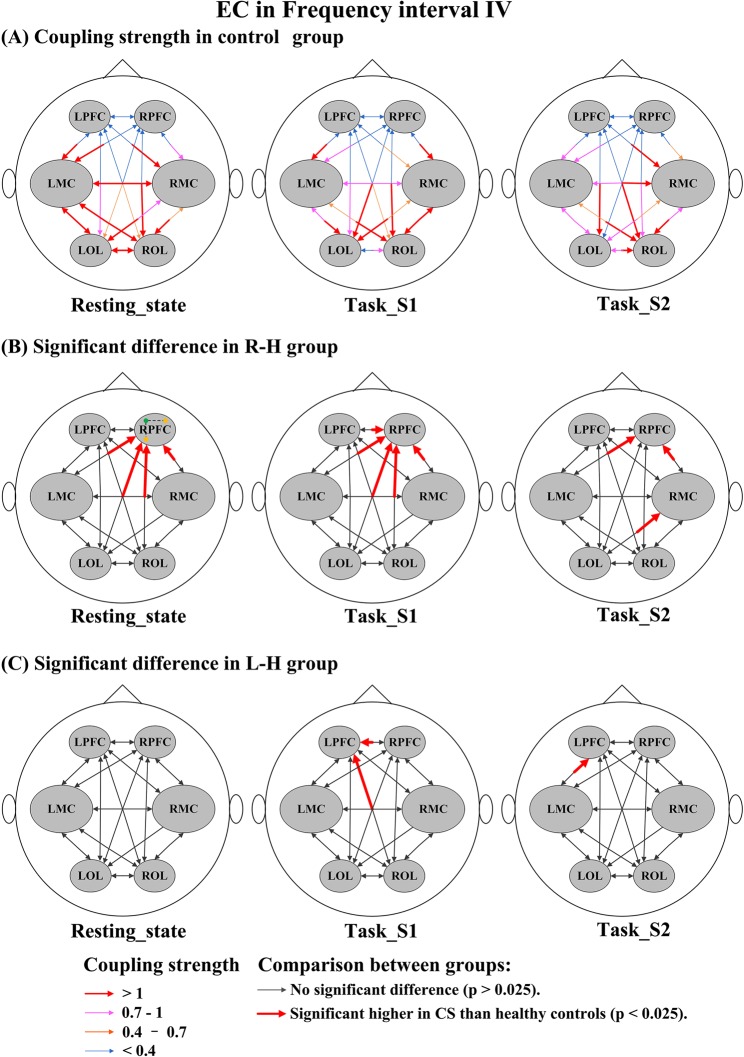
Changes in EC among brain regions for delta HbO_2_ signals in interval IV under different conditions: (**A**) CS in control group under different conditions; Coupling parameters indicate the connection strength and direction, which are coded in the size and color of the arrows. (**B**) Significant difference in R-H group compared with healthy controls; (**C**) Significant difference in L-H group compared with healthy controls.

Figure 7 shows the stroke-related changes in EC among regions for delta HbO_2_ signals in interval I. In task_S1, the CS valves from LPFC→LMC in the R-H group and from RPFC→LPFC, RMC→LPFC, LMC→LPFC, and LPFC→LMC in the L-H group were significantly lower than those in healthy controls. In task_S2, compared with the control group, the CS valves in the R-H group were significantly decreased in LMC→LPFC, RMC→LPFC, and LOL→LPFC and the CS valves in the L-H group were significantly decreased in LPFC→RPFC, LPFC→LMC, LPFC→RMC, LPFC→LOL, RPFC→LPFC, LMC→LPFC, RMC→LPFC, LOL→LPFC, and LMC→LOL. Compared with the healthy controls, the CS valves in stroke patients were significantly decreased in the resting state and task states in the result of delta HHb signals (Fig. S7). In interval II (Fig. 8), in the resting state, the CS valves of connectivity LPFC→LOL (*p* = 0.014) and ROL→LOL (*p* = 0.012) were significantly lower in the R-H group than in the control group. In task_S1 state, the CS valves of connectivity LPFC→LOL (*p* = 0.015) in the R-H group and from LPFC→LMC (*p* = 0.025) in the L-H group showed significant decrease compared with those in the control group. Based on the result of delta HHb signals (Fig. S8), the CS valves in stroke patients were significantly lower in the resting state than those in the control group. In addition, the CS valves in the L-H group were significantly decreased in task_S2 compared with those in healthy controls. In interval III (Fig. 9), in task_S1, the CS in the L-H group was significantly increased in connectivity RMC→LPFC (*p* = 0.023) compared that in healthy controls. In task_S2, the CS valves in L-H group were significantly increased in connectivity LMC→LPFC (*p* = 0.009), RMC→LPFC (*p* = 0.004), and ROL→LPFC (*p* = 0.012) compared those in healthy controls. In the result of delta HHb signals (Fig. S9), the CS values of connectivity to LPFC were significantly increased in the L-H group in the task states compared with those in healthy controls. In interval IV (Fig. 10), in the resting state, the R-H group showed significantly increased CS valves from LMC→RPFC (*p* = 0.007), RMC→RPFC (*p* = 0.021), LOL→RPFC (*p* = 0.010), and ROL→RPFC (*p* = 0.019) compared with those in the control group. In task_S1 state, the CS valves in the R-H group in connectivity LPFC→RPFC (*p* = 0.005), LMC→RPFC (*p* = 0.006), RMC→RPFC (*p* = 0.002), LOL→RPFC (*p* = 0.016), and ROL→RPFC (*p* = 0.003) and the CS valves in the L-H group in connectivity RPFC→LPFC (*p* = 0.022) and ROL→LPFC (*p* = 0.016) were significantly increased as compared with those in control group. In task_S2, the CS valves in the group R-H in connectivity LMC→RPFC (*p* = 0.021), RMC→RPFC (*p* = 0.014), and LOL→RMC (*p* = 0.018) and the CS in the group L-H in connectivity LMC→LPFC (*p* = 0.011) showed significantly increase compared with those in the control group. In this interval, in the result of delta HHb signals (Fig. S10), the CS valves of connectivity to LPFC in the L-H group and the CS valves of connectivity to RPFC in the R-H group were significantly increased compared with those in healthy controls.

## Discussion

In this study, DBI was used to calculate the coupling parameters of delta HbO_2_ and delta HHb signals based on a coupled-phase-oscillator model in different frequency intervals. Measures of cortical activity and frequency-specific EC were used to investigate the effects of stroke on cortical reorganization during a motor rehabilitation task with upper and lower limb linkage. The CS values in intervals I and II were significantly lower in stroke patients than in healthy controls in the resting and task states. Strikingly, the CS values of connectivity to RPFC in interval IV were significantly higher in the R-H group than in the control group in the resting and task states. The L-H group showed significantly higher CS values of connectivity to LPFC in intervals III and IV than the control group during the task state. Similar results were also found for delta HHb signal. This study demonstrated the applicability of fNIRS-based technology to evaluate the effect of rehabilitation tasks on cortical reorganization. Furthermore, this study could serve as a basis for developing novel therapeutic techniques for functional recovery poststroke.

Neurovascular coupling involves the dilation of blood vessels to increase the local blood flow^51,52^. Neurovascular coupling or functional hyperemia initiates increased blood flow and matches the supply of blood and nutrients to the needs following local neuronal activation^53^. As expected from neurovascular coupling, regional glucose consumption in the brain is strongly correlated with local blood flow^54,55^. Neuronal activity, glucose metabolism and blood flow are effectively tied together^56^. The typical fNIRS finding demonstrated an increase of regional cerebral blood flow (rCBF) and regional cerebral oxygen metabolic rate (rCMRO2) during brain activation^57^. The increase in delta HbO_2_ during brain activation showed higher task-induced augmentation of rCBF than that of oxidative metabolism^58^. Usually, the increase of rCBF will exceed the increase of rCMRO_2_, resulting in the increase of delta HbO_2_ and decrease of delta HHb concentrations^59^. Combining the results of delta HbO_2_ and delta HHb, the stroke patients exhibited greater activation in the contralesional motor region during motor rehabilitation task with upper and lower limb linkage. Several neuroimaging studies have demonstrated movement-related overactivation in contralesional motor areas poststroke^60,61^. This present altered pattern of activation in the contralesional and ipsilesional hemispheres during movement was in line with the results of previous studies^6,9^.

In general, the regulation of rCBF is considered a synergistic effect of sympathetic-mediated and local myogenic or metabolic mechanisms. The physiological meaning of the HF oscillations (interval I and interval II), as a part of the systemic circulation, corresponds to cardiac and respiratory activities^62^ that serve as pumps driving blood through the vessels^62^. The effect of the heart pumping and respiratory activities is manifested in the cerebral vessels. In healthy controls, the interactions among the regions in interval I during task_S2 showed significantly increased CS values compared with those in task_S1. In context of the entire duration of the task, increased CS in task_S2 suggests the enhanced regulation of cardiac activity to maintain the rCBF fluctuation within a certain degree in response to the requirements of a task. These increased directed interactions may play an important role in compensating for the fatigue effect. The regulation of rCBF is an integrative process during which cardiovascular function plays an important role^63^. Stroke leads to the degeneration of the vascular system^64^. Changes in the vascular system may influence the regulation of rCBF according to the task and lead to impaired cardiovascular systems in stroke. Combining the results of delta HbO_2_ and delta HHb signals, the stroke patients showed significantly lower EC (p < 0.025) in intervals I and II in the resting and task states compared with the healthy controls. The stroke-related decrease in CS among regions in interval I may be evidence of impaired cardiovascular regulation poststroke.

As a component of systemic activity, modulation of respiratory activity in this frequency interval also plays a role in adjusting the rCBF according to the task. The MCs are reportedly involved in the attention, execution, and planning of voluntary movements^65^. The OL is essential for processing visual information and plays a crucial role in the coordination of language, perception, and abstraction^66,67^. In addition to the movement of the upper and lower extremities, motor rehabilitation also involves other modalities, such as cognitive or visual processing. The significantly increased interactions between the MC and OL in response to movement revealed the modulation of brain networks by the rehabilitation task in this frequency interval. The influence exerted on the LOL in interval II significantly decreased in the patients with right-hemiparesis in the resting state compared with the healthy controls. The decreased directed interactions in the brain network in this interval might be partly attributed to vessel stiffness in stroke patients.

The LF oscillations of delta HbO_2_ signals in interval III have been suggested to originate locally from the intrinsic myogenic activity of smooth muscle cells in resistance vessels^68–71^. Vascular smooth muscles can contract or relax in response to an increase or decrease in intravascular pressure^72,73^. This process is known as the myogenic autoregulation mechanism, through which rCBF is modulated to a certain degree. Spontaneous LF oscillations of delta HbO_2_ strongly decreased in the patients with cerebral microangiopathy^74^. Wavelet analysis has shown that the amplitude of myogenic oscillations is decreased in the elderly^71^. In interval III, compared with the healthy controls, the stroke patients had relatively less significant changes in the EC network induced by the motor rehabilitation task with upper and lower limb linkage. The reduced cerebrovascular response in this interval suggests that the myogenic regulatory mechanism contributes relatively less to the regulation of cerebral blood perfusion in response to the rehabilitation task in stroke patients. The results in this interval may suggest the reduced contractility of the smooth muscle layer of the arteriole and increased vessel stiffness with stroke^75^. Analysis of the results for each type of signal showed that patients with left-hemiparesis showed significantly increased directed influence on the contralesional PFC during the movement task compared with the healthy controls. These findings suggest that plastic reorganization compensated for motor network impairment in the ipsilesional hemisphere. The brain after stroke must therefore recruit these contralesional regions more than would be normally necessary to perform the same task^76^. Limb linkage rehabilitation training can stimulate the coordination of the upper and lower limbs, trunk stability and balance control ability. The PFC modulates most human behavior and executive control processes^77^, and reportedly influences balance control during locomotion^78^. In this case, the result obtained was consistent with the conclusion that the stroke patients invoke greater cognitive control when performing relatively simple tasks^79^.

The oscillations of frequency interval IV, which are related to neurogenic activity of vessels, reflected the involvement of neurogenic control^62^. The autonomous nervous system participates in vasoconstriction by regulating the release of substances that affect the activities of smooth muscles^80^. This process can lead to changes in the vessels’ radii and resistance. Thus, the continuous activity of the autonomous nervous system serves to maintain the basal level of contraction of the vessels. Age-related attenuation of the cerebrovascular response was observed in this frequency interval^71^. The most important finding in this interval was the stroke-related changes in the directed interactions among the regions. Analysis of the results for each type of signal found a significant increase in the directed influence on the contralesional PFC in stroke patients. Functional recovery from stroke is now widely believed to be a result of central nervous system reorganization^81^. Additionally, contralesional recruitment plays an integral role in motor recovery poststroke^2^. Furthermore, increased recruitment of the contralesional PFC may be helpful in reinforcing the management of cognitive load required for motor performance^31^. In this study, recruitment of and adaptation in contralesional PFC may help patients achieve the best results possible given their anatomical constraints. Taken together, the stroke-related changes in the EC pattern appear to reflect abnormalities of brain network interactions and plastic reorganization in response to the specific motor rehabilitation task.

## Limitations

The present study has several potential limitations. The first limitation is the small sample size and large difference in time poststroke among the participants. The stroke patients who participated in this study were classified into left and right hemiparesis groups. The time since stroke, severity of deficit at baseline, site of lesion, and other biological factors (e.g., age of patients) most likely contribute to inter-individual differences. The task may also be experienced as more or less requiring effort depending on each patient’s degree of recovery. These conditions might be reflected in different reorganization patterns in different patients. In the future, additional participants need to be recruited to improve understanding of cortical reorganization in stroke patients. The second limitation in this study is related to the use of fNIRS as a functional imaging tool for the measurement of cerebral hemodynamic changes. Physiological noise originating from superficial tissue layers (scalp and skull) can inevitably contaminate the raw fNIRS signals. In the present study, wavelet transform was applied to distinguish characteristic frequency intervals of cerebral NIRS signals corresponding to specific origins. The key point of this study is to investigate the effect of stroke on cortical reorganization in responding to the rehabilitation task based on different physiological meanings. Third, the relatively low spatial resolutions of fNIRS measurement complicated the investigation on the precise recruitment and adaption of each cortical area in cortical reorganization for the rehabilitation task. In this study, we focused on stroke-related changes in large-scale connectivity networks, including the PFCs, MCs, and OLs. More precise cerebral location of fNIRS channels may obtain more detailed information about functional reorganization of the brain. Further studies combined with other neuroimaging methodologies, such as fMRI, are required to elucidate the neurological mechanism for poststroke rehabilitation.

## Conclusions

In this study, frequency-specific EC was calculated by using coupling functions and DBI based on a coupled-phase-oscillator model to investigate a motor rehabilitation task-related changes in EC in stroke patients. Stroke patients exhibited greater activation in contralesional than in ipsilesional motor regions during a motor rehabilitation task, thus indicating an asymmetric cortical activation pattern in stroke survivors versus healthy controls. The significantly increased CS on the contralesional PFC in the low frequency band suggested that plastic reorganization of cognitive resources was used to compensate for impaired function in stroke patients during the rehabilitation task. Assessing the frequency-specific EC based on fNIRS signals clarified the task-related reorganization of brain functional networks and could help develop novel assessment techniques for rehabilitation.

## Supplementary information

Supplement
