# Supplementary material for: Limb linkage rehabilitation training-related changes in cortical activation and effective connectivity after stroke: A functional near-infrared spectroscopy study
Source: Sci Rep. 2019 Apr 17;9:6226. doi: 10.1038/s41598-019-42674-0 (PMC6470232; doi:10.1038/s41598-019-42674-0)
Supplement: Supplementary file 1 — Supplement [file 41598_2019_42674_MOESM1_ESM.pdf]

Limb linkage rehabilitation training-related changes in cortical activation and effective connectivity after stroke: A functional near-infrared spectroscopy study

Congcong Huo[1#], Gongcheng Xu[2#], Zengyong Li[1,4*], Zeping Lv[3], Qianying Liu[2], Wenhao Li[2], Hongzhuo Ma[3], Daifa Wang[2,5*], Yubo Fan[1,2,5*]

1. Beijing Key Laboratory of Rehabilitation Technical Aids for Old-Age Disability, National Research Center for Rehabilitation Technical Aids, Beijing, 100176

2. Key Laboratory for Biomechanics and Mechanobiology of Ministry of Education, School of Biological Science and Medical Engineering, Beihang University, 100086, Beijing, China

3. Rehabilitation Hospital, National Research Center for Rehabilitation Technical Aids, Beijing, 100176

4. Key Laboratory of Rehabilitation Aids Technology and System of the Ministry of Civil Affairs, Beijing, 100176

5. Beijing Advanced Innovation Center for Biomedical Engineering, Beihang University, Beijing, 100083

Contact Information

*Corresponding author:

Zengyong Li

National Research Center for Rehabilitation Technical Aids, Beijing, 100176, P. R. China

Tel: +86-10-58122886, Email: lizengyong@nrcrta.cn

Daifa Wang

School of Biological Science and Medical Engineering, Beihang University Beijing, 100176

Email: daifa.wang@buaa.edu.cn

Yubo Fan

National Research Center for Rehabilitation Technical Aids, Beijing, 100176, P. R. China

Email: fanyubo@nrcrta.cn

# These authors contributed equally to this work and should be considered co-first authors.

Supplement 1

Supplemental Methods and Materials

Data Preprocessing

In this study, we used a six-order Butterworth band-pass filter to obtain the filtered signals of 0.021-2Hz with an improved signal-to-noise ratio. **Figure S1 and S2** show the filter response and the zero-pole map. All poles of the filter were in the unit circle, which indicated that the filter is stable.

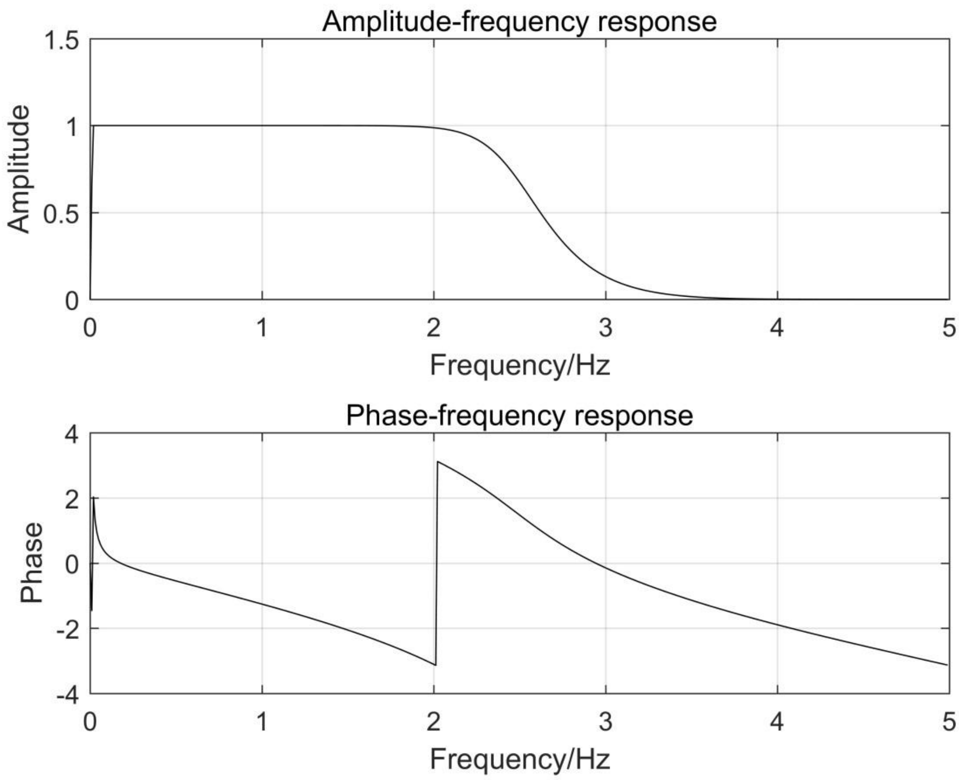

Fig S1. The filter response of the Butterworth filter.

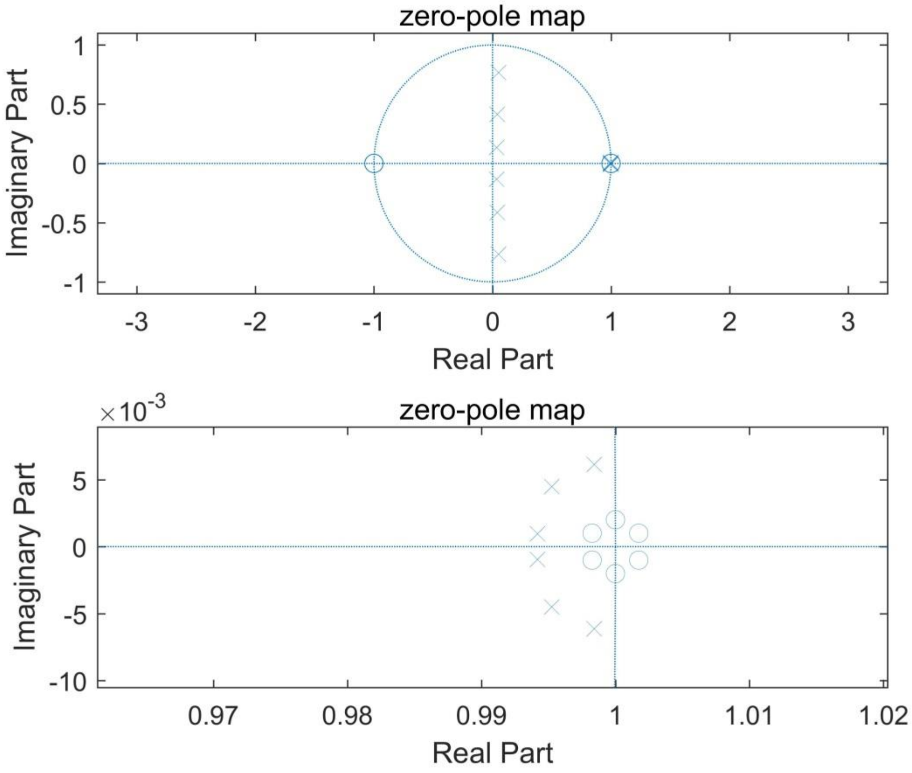

Fig S2. The zero-pole map of the Butterworth filter. All poles of the filter were in the unit circle.

Wavelet transform

Continuous wavelet transform is mainly used to extract the feature of signal and is a common tool for analyzing localized intermittent oscillations in a time series[1]. In choosing the wavelet function, there are several factors to consider[2]:

(1) The use of an orthogonal wavelet function implies the use of the discrete wavelet transform. Nonorthogonal wavelet function can be used with either the discrete or the continuous wavelet transform. Generally, smooth and continuous variations in wavelet amplitudes can be obtained by using nonorthogonal transform in time series analysis.

(2) A complex wavelet function will return information about both amplitude and phase and is better adapted for capturing oscillatory behavior.

(3) A wavelet can be characterized by how localized it is in time (Δt) and frequency (Δw). The classical version of the Heisenberg uncertainty principle tells us that there is always a tradeoff between localization in time and frequency. The time (Δt) and frequency (Δw) resolutions are connected by $\Delta t \Delta w \geq c$, where c is a constant. This equality is attained only for a Gaussian function[3].

Morlet mother wavelets belong to Nonorthogonal wavelet function and it is a complex plane wave multiplied by a Gaussian envelope function[4]. The rationale for using the Morlet wavelet is that its Gaussian envelope provides good localization of events in both time and frequency. It is defined as:

$$\psi_0(t) = \pi^{-1/4} e^{iw_0 t} e^{-t^2/2}$$

Where w_0 is dimensionless frequency and t is dimensionless time. Morlet wavelet-based transform can detect these oscillations with logarithmic frequency resolution, follow the variations of their frequencies and amplitudes in time[5,6], and provide a good balance between time and frequency localization[1]. In this work, the wavelet coefficients obtained using the complex Morlet wavelet are complex numbers which define the amplitude and instantaneous phase for each frequency and time[3].

Surrogate test

Generation of surrogate signals

Amplitude adjusted Fourier transform (AAFT) surrogates are generated by a phase randomization process, preserving the power spectrum and the amplitude distribution[7]. The procedure for AAFT surrogates is as follows[8]:

1. Sort the original time series s in ascending order, and rank the original location of the values from

1 to L, where L is the length of the time series. For example, if s_n is the fifth smallest element among all s, $ranks_n$ will be 5.

2. Generate a Gaussian noise signal with the length L, and sort in ascending order to give Gs.

3. Reorder Gs to match the ranks of the original time series to create a new vector x. If s_n is the n th smallest of all s, then x_n will be the n th smallest of all Gs. Therefore, $x_{n=1,......L} = Gs_{ranka(1,......L)}$.

4. Calculate the Fourier transform ft_x of the signal x_n.

5. Generate a vector of random phases φ_r, with length $L/2$.

6. As the Fourier transform is symmetrical, to create the new phase randomized vector ft_r, multiply the first half of ft_x by exp $(i\varphi_r)$ to create the first half of ft_r. The remainder of ft_r is then the horizontally flipped complex conjugate of the first half.

7. Finally, the inverse Fourier transform of ft_r gives the surrogate of x, denoted xs.

8. Sort xs in ascending order, and again assign a rank to these values in a new vector *ranks*2.

9. Reorder the sorted values of the original signal according to *ranks*2 to obtain the surrogate.

Supplemental Results

Cortical activation patterns during the rehabilitation task

Figure S3 shows cortical activation patterns with respect to delta HHb signal in healthy and stroke participants during the resting state, task_S1, and task_S2. The letters indicate the positions of the light source and detector, and a pair of adjacent light sources and detectors forms a channel. In a given state, the average value of the delta HbO_2 concentration change (over 10 min) at the channel midpoint represents its change in the channel region, from which an image is generated by

interpolating the inverse distance. The color bar number range on the right specifies the color depth.

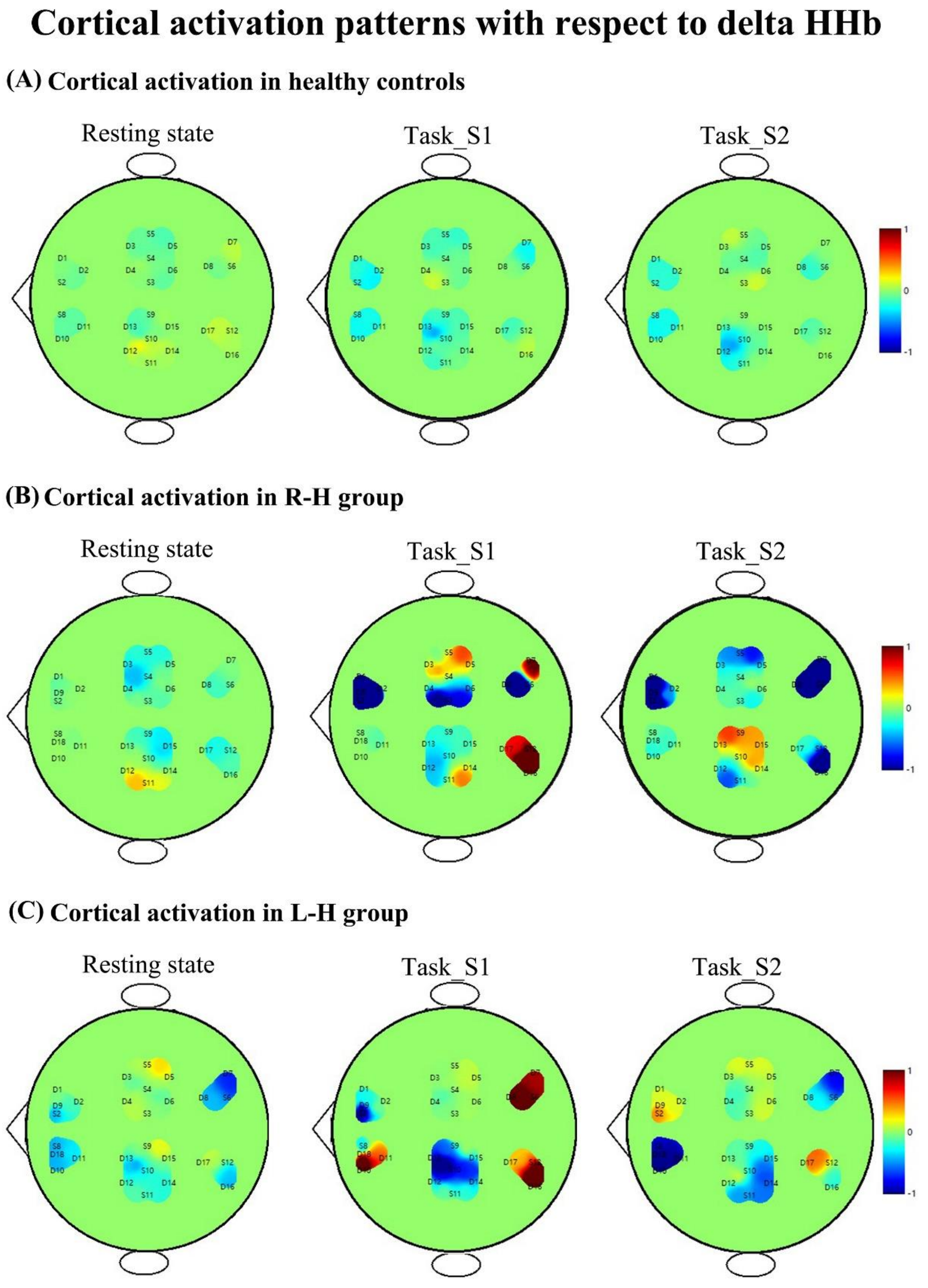

Fig S3. Cortical activation patterns with respect to delta HHb during different conditions in participants (A) healthy control; (B) patient with right-hemiplegia; and (C) patient with left-hemiplegia.

Effective connectivity

Changes in EC from resting state to task state

Figure S4 shows the significant changes in frequency-specific EC with respect to delta HHb signals from the resting to task states in the healthy controls. Significant increased CS values among the regions were found in task states compared with resting state in high-frequency bands (interval I and II). The CS values in intervals III and IV significantly decreased in task states as compared with the resting state. **Figure S5** shows the state-related changes in frequency-specific EC with respect to delta HHb signals in the R-H group. In interval I, compared with the resting state, the CS values were significantly increased in LMC→LOL (p = 0.015) in task_S1 and in LOL→RPFC (p = 0.011) in task_S2. In interval II, compared with the resting state, the CS values were significantly increased in LOL→RPFC (p = 0.014) in task_S1 and in LMC→LPFC (p = 0.009) in task_S2. In interval III, compared with the resting state, the CS was significantly decreased in RMC→LPFC (p = 0.009) in task_S2. **Figure S6** shows the state-related changes in frequency-specific EC with respect to delta HHb signals in the L-H group. In interval II, compared with the resting state, the CS was significantly increased in LOL→RMC (p = 0.015) in task_S2. The CS was in ROL→RMC (p = 0.002) significantly decreased in task_S2 compared with task_S1.

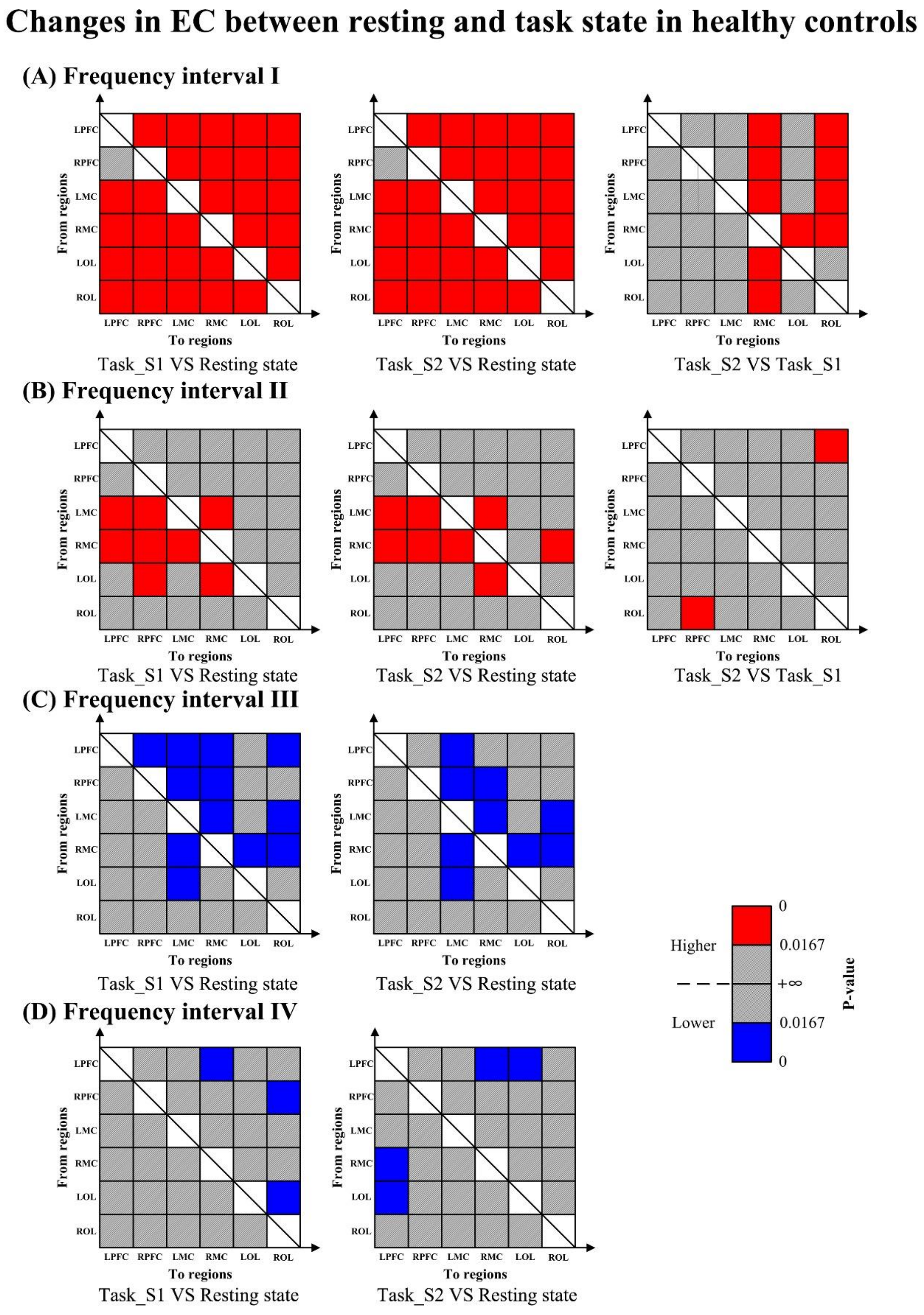

Fig S4. Changes in frequency-specific EC for delta HHb signals of the healthy controls between different states: (A) Comparison in frequency interval I; (B) Comparison in frequency interval II; (C) Comparison in frequency interval III; (D) Comparison in frequency interval IV.

Changes in EC between resting and task state in R-H group

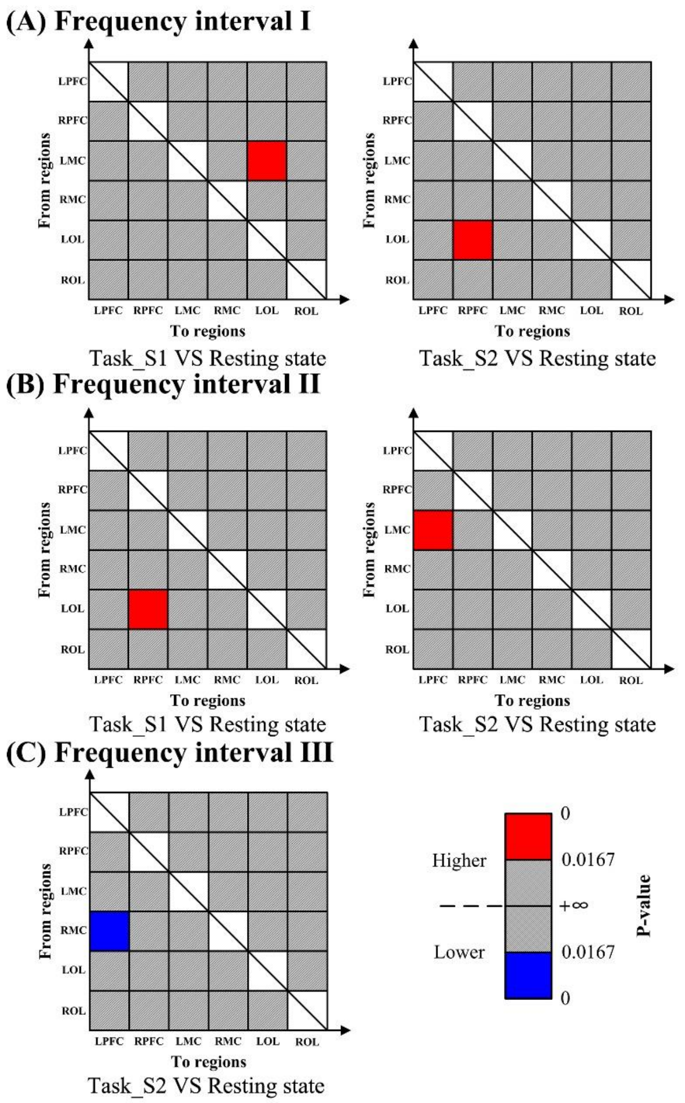

Fig S5. Changes in frequency-specific EC for delta HHb signals of the R-H group between different states: (A) Comparison in frequency interval I; (B) Comparison in frequency interval II; (C) Comparison in frequency interval III.

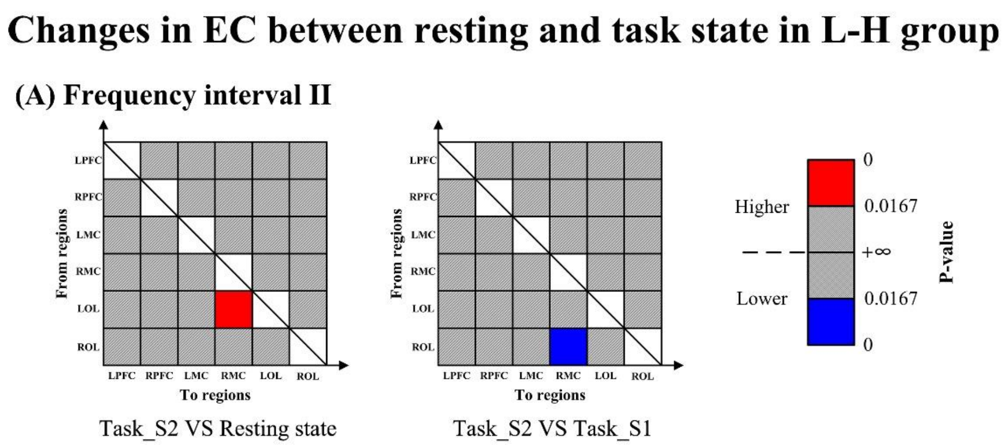

Fig S6. Changes in frequency-specific EC for delta HHb signals of the L-H group between different states: (A) Comparison in frequency interval II.

Comparison in EC between healthy participants and stroke patients under different conditions

Figure S7 shows the stroke-related changes in EC among regions for delta HHb signals in interval I. In resting state, the CS values from RPFC→LOL (p = 0.023) and RPFC→ROL (p = 0.008) in the L-H group and from ROL→LOL (p = 0.004) in the R-H group were significantly lower than those in healthy controls. The CS values from LOL→RMC (p = 0.009) in the L-H group and from ROL→RPFC (p = 0.024) in the R-H group were significantly increased in the resting state as compared with healthy controls. In task_S1, the CS values from LPFC→LMC (p = 0.004) and LPFC→LOL (p = 0.014) in the L-H group and from LPFC→LMC (p = 0.018) and RPFC→LMC (p = 0.023) in the R-H group were significantly lower than those in healthy controls. In task_S2, compared with that in the control group, the CS values in the L-H group were significantly decreased in LPFC→RPFC (p = 0.002), LPFC→LMC (p = 0.0005), LPFC→RMC (p = 0.0004), LPFC→LOL (p = 0.01), RPFC→LMC (p = 0.024), LMC→RPFC (p = 0.014), RPFC→RMC (p = 0.015), RMC→RPFC (p = 0.023) and the CS values in the R-H group were significantly decreased in LPFC→LMC (p = 0.011), LPFC→RMC (p = 0.02), LPFC→ROL (p = 0.009), RPFC→LMC (p =

0.02), RPFC→RMC (p = 0.01), RPFC→ROL (p = 0.006) and RMC→ROL (p = 0.017). In interval II **(figure S8)**, in the resting state, the CS values of LPFC→LOL (p = 0.001) and ROL→RPFC (p = 0.013) in L-H group and of connectivity ROL→LOL (p = 0.007) were significantly lower than those in the control group. In task_S2 state, the CS of connectivity LPFC→ROL (p = 0.012) in the L-H group showed significant decrease compared with that in the control group. In interval III **(figure S9)**, compared with control group, the CS values from LPFC→LMC (p = 0.007), RPFC→LMC (p = 0.002), RPFC→RMC (p = 0.021), LMC→RMC (p = 0.024), RMC→LMC (p = 0.007) and LOL→LMC (p = 0.013) in resting state, from LOL→LMC (p = 0.017) in task_S1 state and from RPFC→LMC (p = 0.024) in task_S2 state were significantly decreased in L-H group. However, the CS values from LOL (p = 0.007), ROL (p = 0.018), LMC (p = 0.007) and RMC (p = 0.007) to LPFC in task_S1 state and from LMC (p = 0.015), RMC (p = 0.006) and ROL (p = 0.024) to LPFC in task_S2 state were significantly higher than those in healthy controls. In resting state, the CS values in the R-H group were significantly increased in LMC→LPFC (p = 0.007), RMC→LPFC (p = 0.002) and LOL→LPFC (p = 0.02) compared those in healthy controls. In interval IV **(figure S10)**, compared with controls group, the CS values from LPFC→LOL (p = 0.016) and LPFC→RMC (p = 0.024) in resting state were significantly decreased in L-H group. The CS values from LMC→LPFC (p = 0.019), ROL→RPFC (p = 0.024) and RMC→RPFC (p = 0.005) in resting state, from LMC (p = 0.005), RMC (p = 0.003) and RPFC (p = 0.02) to LPFC in task_S1 state and from RPFC (p = 0.0004), LMC (p = 0.009), RMC (p = 0.002) and LOL(p = 0.006) to LPFC in task_S2 state in L-H group were significantly higher than those in healthy controls. In task_S1, the CS values in the R-H group were significantly increased in connectivity LPFC→RPFC (p = 0.005), LMC→RPFC (p = 0.016), RMC→RPFC (p = 0.002), ROL→RPFC (p = 0.024) and LOL→RPFC (p = 0.016) compared with

those in healthy controls.

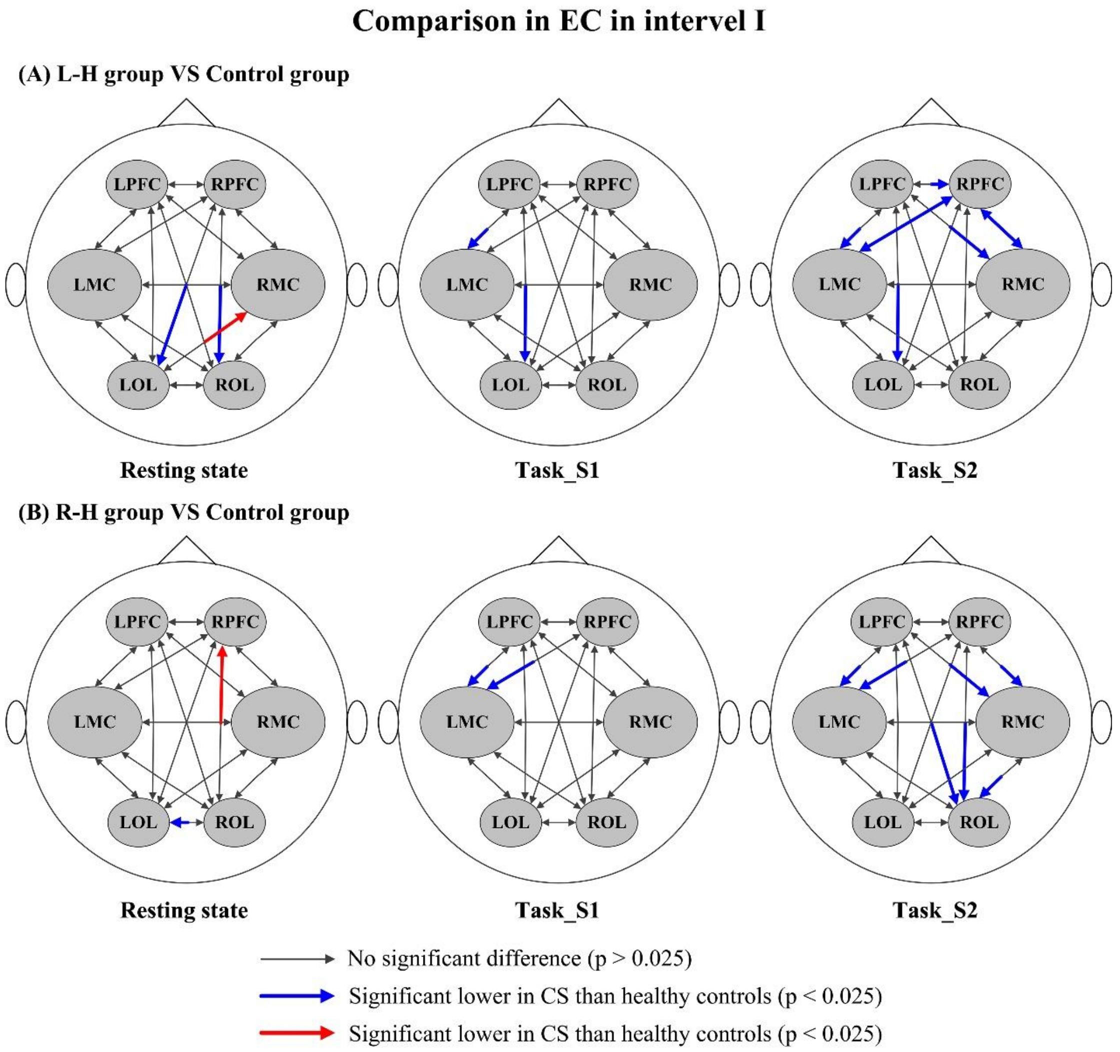

Fig S7. Stroke-related changes in EC among brain regions for delta HHb signals in interval I under different conditions: (A) Significant difference between L-H group and healthy controls; (B) Significant difference between R-H group and healthy controls.

Comparison in EC in intervel II

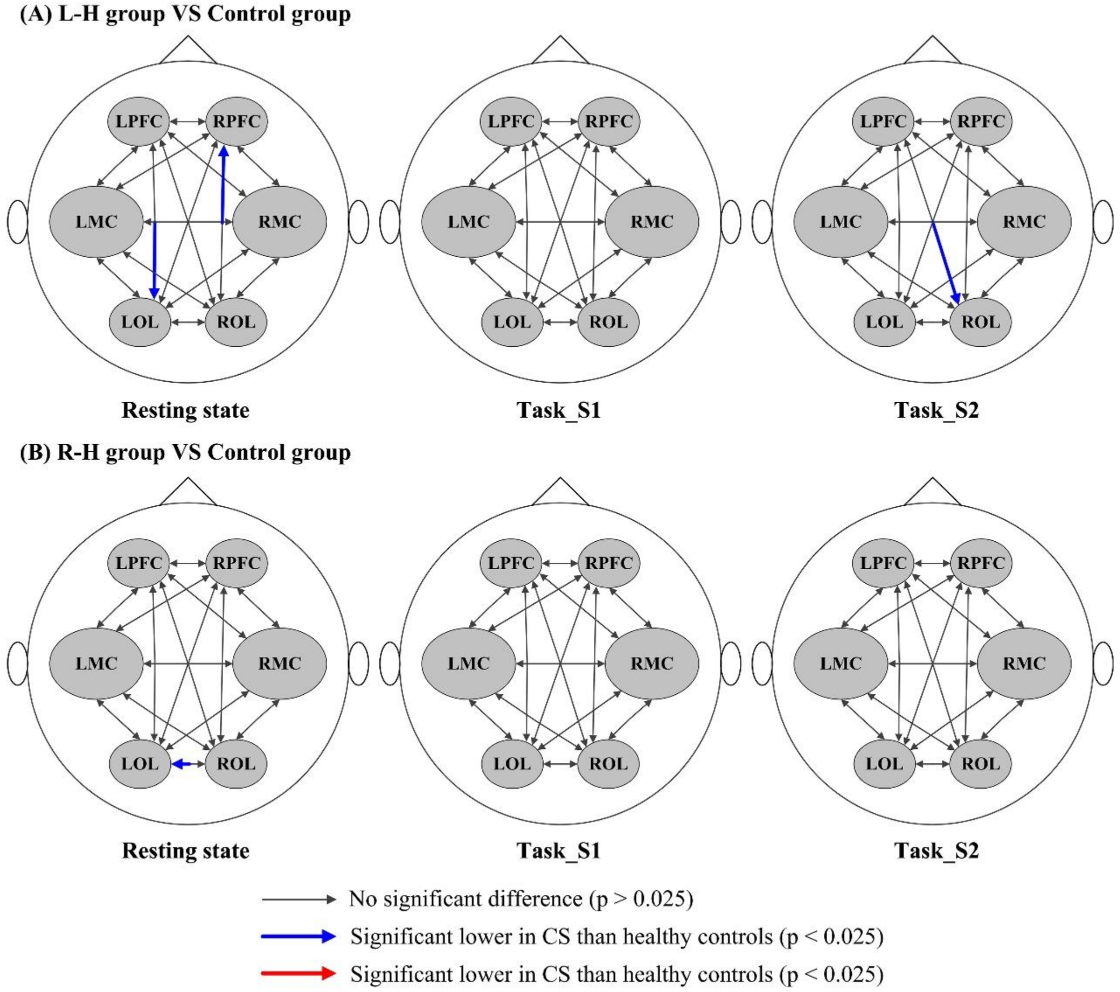

Fig S8. Stroke-related changes in EC among brain regions for delta HHb signals in interval II under different conditions: (A) Significant difference between L-H group and healthy controls; (B) Significant difference between R-H group and healthy controls.

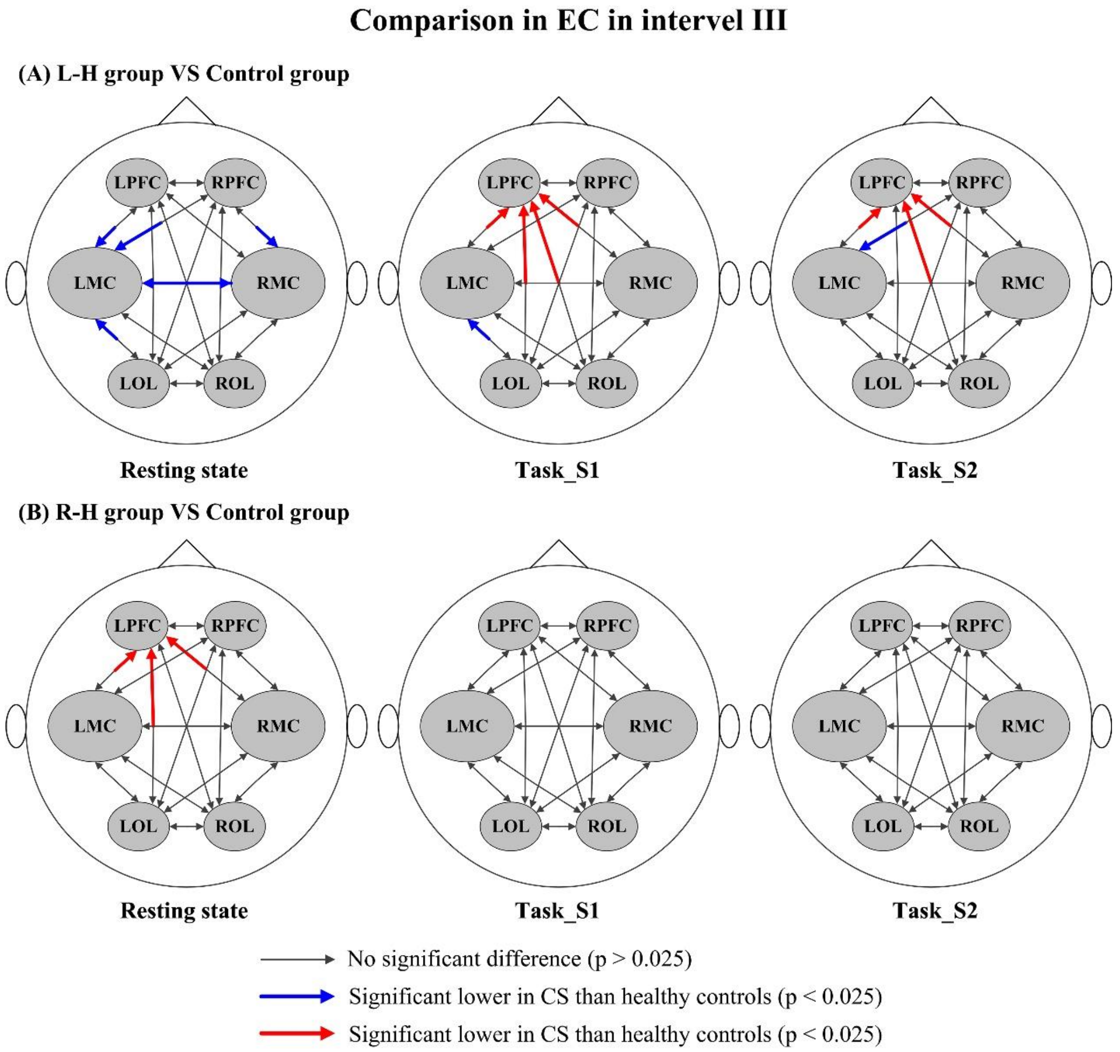

Fig S9. Stroke-related changes in EC among brain regions for delta HHb signals in interval III under different conditions: (A) Significant difference between L-H group and healthy controls; (B) Significant difference between R-H group and healthy controls.

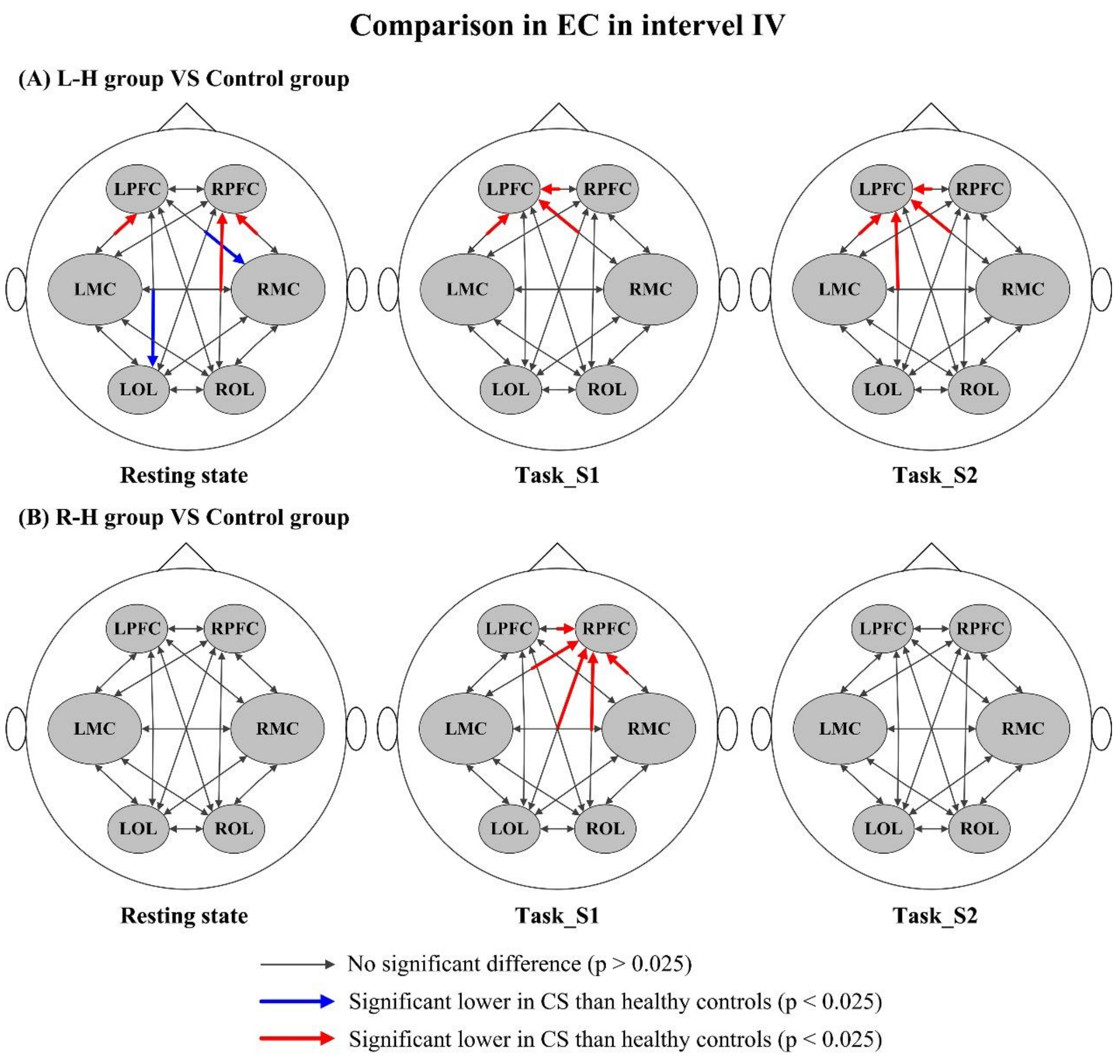

Fig S10. Stroke-related changes in EC among brain regions for delta HHb signals in interval IV under different conditions: (A) Significant difference between L-H group and healthy controls; (B) Significant difference between R-H group and healthy controls.

Supplemental Reference

1 Grinsted, A., Moore, J. C. & Jevrejeva, S. Application of the cross wavelet transform and wavelet coherence to geophysical time series. *Nonlinear Processes in Geophysics,11,5/6(2004-11-18)* **11**, 561-566 (2004).

2 Henderson-Sellers, A. *et al.* Tropical Cyclones and Global Climate Change: A Post-IPCC Assessment. *Bulletin of the American Meteorological Society* **79**, 19-38 (1998).

3 Kilmer, W. A Friendly Guide To Wavelets. *Physics Today* **48**, 57-58 (1995).

4 Goupillaud, P., Grossmann, A. & Morlet, J. Cycle-octave and related transforms in seismic signal analysis. Geoexploration 23(1):85-102. *Geoexploration* **23**, 85-102 (1984).

5 Stefanovska, A., Bracic, M. & Kvernmo, H. D. Wavelet analysis of oscillations in the peripheral blood circulation measured by laser Doppler technique. *IEEE Transactions on Biomedical Engineering* **46**, 1230-1239 (1999).

6 AnetaStefanovska. Physics of the human cardiovascular system. *Contemporary Physics* **40**, 31-55 (1999).

7 Theiler, J., Eubank, S., Longtin, A., Galdrikian, B. & Farmer, J. D. Testing for nonlinearity in time series: the method of surrogate data. *Physica D-nonlinear Phenomena* **58**, 77-94 (1992).

8 Lancaster, G., Iatsenko, D., Pidde, A., Ticcinelli, V. & Stefanovska, A. Surrogate data for hypothesis testing of physical systems. *Physics Reports* (2018).
